# Olanzapine affects bone formation via oral *Enterococcus* through *SAA1* gene and extracellular matrix-related pathways

**DOI:** 10.3389/fcimb.2025.1673931

**Published:** 2026-01-06

**Authors:** Hui Yuan, Min-Yuan Wang, Rui-Xi Liu, Senjeet Sreekissoon, Qiong Liu, Li Tan, Ya-Qiong Zhao, Meng-Mei Zhong, Qian Zhang, Xiao-Lin Su, Ning-Xin Chen, Mei Wang, Yi-Fan Yang, Jian-Nan Li, He-Qiong Zheng, Jin-Dong Chen, Yun-Zhi Feng, Feng-Yi Zhang, Yue Guo

**Affiliations:** 1 Department of Stomatology, Hunan Provincial Engineering Research Center of Digital Oral and Maxillofacial Defect Repair, Hunan Provincial Clinical Research Center for Oral Diseases, The Second Xiangya Hospital, Central South University, Changsha, Hunan, China; 2 Hunan Key Laboratory of Oral Health Research, Xiangya Stomatological Hospital, Xiangya School of Stomatology, Central South University, Changsha, Hunan, China; 3 Department of Psychiatry, National Clinical Research Center for Mental Disorders, National Center for Mental Disorders, The Second Xiangya Hospital, Central South University, Changsha, Hunan, China

**Keywords:** olanzapine, oral *Enterococcus*, bone formation, serum amyloid A1 gene, extracellular matrix

## Abstract

**Background:**

Olanzapine is a commonly used drug in the treatment of schizophrenia, but the mechanism of abnormal bone metabolism caused by olanzapine is still unclear. The change of microflora may be an important factor leading to the change of bone metabolism. Therefore, the purpose of this study was to explore a plausible hypothesis that olanzapine may aggravate abnormal bone metabolism and cause bacterial imbalance in patients with schizophrenia.

**Methods:**

This study intervened in mice by gavage with olanzapine to detect changes in alveolar bone tissue and oral microbiota. The effect of related bacteria on osteogenesis was further examined.

**Results:**

The results showed that *Enterococcus* increased, the bone mass and type I collagen of alveolar bone decreased. *Enterococcus* lipoteichoic acid (LTA) inhibited osteogenic differentiation and up-regulated *SAA1* gene expression. *SAA1* gene can down-regulate the expression of *COL1A1* gene, and the proteins encoded by the two may interact.

**Conclusions:**

Olanzapine may increase the relative abundance of oral *Enterococcus*, whose components are plausibly linked to increased expression of *SAA1* gene and inhibition of bone formation through extracellular matrix-related pathways. These exploratory findings support further exploration of microbiota-based strategies to alleviate skeletal complications and promote oral health. The clinical research presented in this paper has been registered on ClinicalTrials.gov, a platform of the U.S. National Institutes of Health (Registration Number: NCT06123897; URL: https://clinicaltrials.gov/ct2/show/NCT06123897), with the registration date of November 9, 2023.

## 1 Introduction

With increasing social pressure, the incidence of psychiatric diseases such as schizophrenia has risen significantly (Charlson et al., 2018; Jauhar et al., 2022). As one of the top ten causes of disability worldwide, schizophrenia has placed a substantial economic burden on society. Atypical antipsychotic drugs are currently the most common treatment for schizophrenia, and olanzapine is one of the most widely used among them. It has minimal extrapyramidal reactions and anticholinergic effects. As a dopamine-serotonin antagonist, olanzapine binds with high affinity to the 5-hydroxytryptamine receptor 2A (5-HT2A) and effectively alleviates psychotic symptoms in patients. However, the use of olanzapine can further exacerbate abnormal bone metabolism in schizophrenia patients (Maagensen et al., 2021), with up to two-thirds of patients experiencing impaired bone mineral density after taking the drug (De Hert et al., 2016; Sabe et al., 2022). Therefore, the impact of olanzapine on bone metabolism cannot be overlooked.

Atypical antipsychotics can worsen bone metabolism through various pathways. The prevailing view is that antipsychotics primarily cause abnormal bone metabolism by up-regulating prolactin levels (Naidoo et al., 2003). However, some studies have found that patients with schizophrenia do not always exhibit significant changes in serum prolactin levels after taking olanzapine, suggesting that olanzapine may induce abnormal bone metabolism through non-hyperprolactinemic pathways. Further research is required to explore the mechanisms by which olanzapine affects bone formation in schizophrenia.

Microbiota has been shown to be closely linked to bone formation. More than half of the bacteria in the human body are colonized in the gastrointestinal tract (29%) and oral cavity (26%) (Peterson et al., 2009). Both the intestinal and oral microbiota have been proven to influence bone metabolism (Lu et al., 2021; Zhao et al., 2022; Duffuler et al., 2024; Xu et al., 2024), and changes in intestinal microbiota may also affect the oral microbiota (Park et al., 2021). Additionally, it has been reported that atypical antipsychotic drugs possess certain antibacterial properties and can cause disturbances in the intestinal microbiota (Nehme et al., 2018; Chait et al., 2020; Chen et al., 2023), although there are few studies focusing on the oral microbiota. The changes in oral microbiota following olanzapine administration and their effects on bone metabolism require further investigation.

Therefore, this study aims to investigate the clinical phenomenon of olanzapine exacerbating abnormal bone metabolism and causing microbiota imbalances in schizophrenia patients. This will help explore the role and mechanisms of oral microbiota dysbiosis, providing new therapeutic targets and research directions for addressing abnormal bone metabolism in schizophrenia patients after medication use.

## 2 Results

### 2.1 Olanzapine treatment results in a significant decrease in bone mass

**Figure 1:**
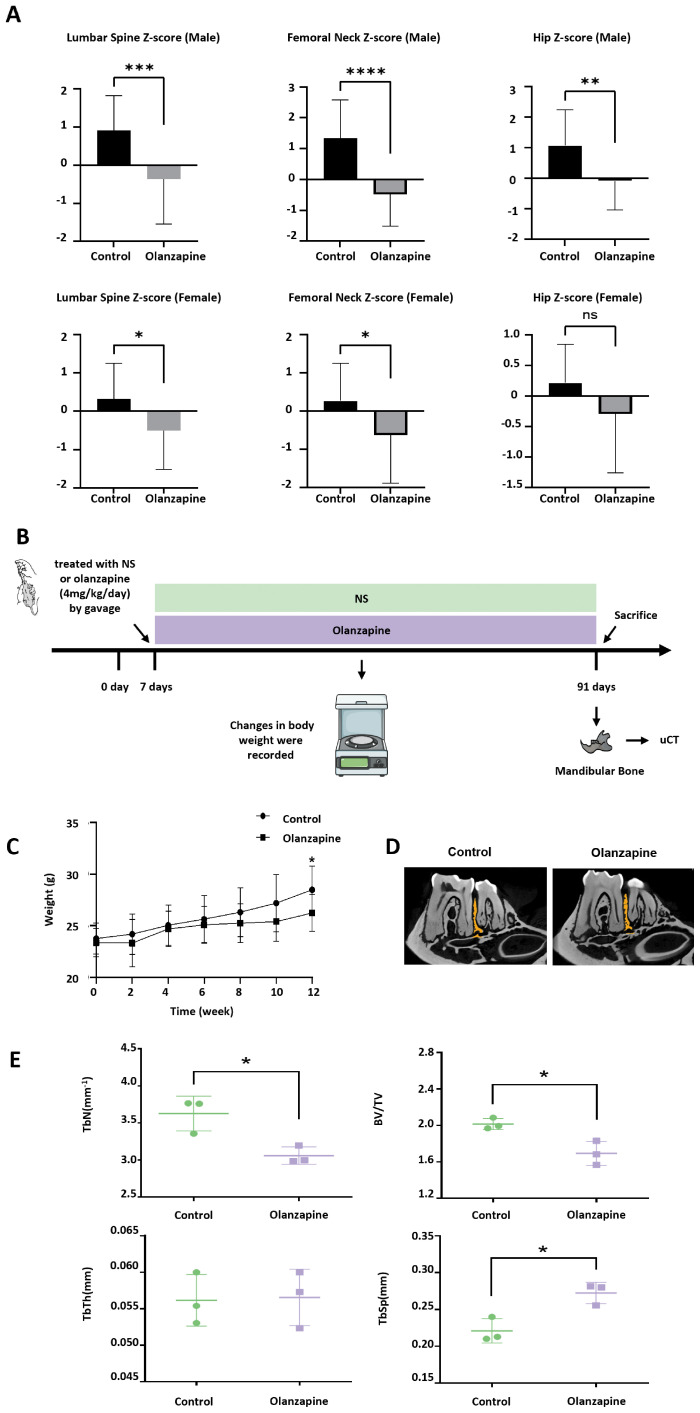
Clinical and animal studies, including bone mineral density (BMD) assessment, olanzapine intervention mouse model construction, and analysis of body weight and bone mass. **(A)** Z-scores of lumbar spine, femoral neck, and hip BMD in male and female patients with schizophrenia receiving long-term olanzapine treatment and in healthy controls. **(B)** Flowchart of mouse model construction and subsequent experimental procedures. **(C)** Body weight changes in control and olanzapine-treated mice during the 12-week experiment. **(D)** Representative micro-CT images of mandibular bone in mice. **(E)** Quantitative analysis of bone parameters from micro-CT, including trabecular number (TbN), bone volume fraction (BV/TV), trabecular thickness (TbTh), and trabecular separation (TbSp). Data are presented as mean±SD. Statistical analysis was performed using Student’s t-test. Significance levels: ns, not significant; *P*<0.05 (*); *P*<0.01 (**); *P*<0.001 (***); *P*<0.0001 (****).

In the clinical study, male patients taking olanzapine had significantly lower Z-scores for the lumbar spine (–0.36 vs. 0.91), femoral neck (–0.49 vs. 1.34), and hip (–0.09 vs. 1.06) than controls (*P*<0.05). In females, Z-scores for the lumbar spine (–0.51 vs. 0.32) and femoral neck (–0.63 vs. 0.27) were also lower (*P*<0.05), with a trend toward reduction at the hip (–0.30 vs. 0.21, *P*=0.092) (**Figure 1A**, **Tables 1**, **2**). In the overall sample (pooled across sex), unadjusted linear regression showed that olanzapine use was associated with lower BMD Z-scores at all skeletal sites: femoral neck (β=−1.16, 95% CI −1.67 to −0.65; *P*<0.001), lumbar spine (β=−0.94, 95% CI −1.43 to −0.46; *P*<0.001), and hip (β=−0.72, 95% CI −1.17 to −0.27; *P*=0.003). After multivariable adjustment for covariates, the associations remained statistically significant and were slightly stronger: femoral neck (β=−1.34, 95% CI −1.84 to −0.83; *P*<0.001), lumbar spine (β=−1.04, 95% CI −1.50 to −0.57; *P*<0.001), and hip (β=−0.88, 95% CI −1.33 to −0.43; *P*<0.001) (**Supplementary Table S1**). These findings suggest bone mass loss in patients taking olanzapine. Therefore, we subsequently explored this hypothesis through *in vivo* animal experiments and *in vitro* cell experiments. As shown in **Figure 1B**, 3-month-old mice were administered normal saline or olanzapine via gavage for 12 weeks, during which the weight of the mice was recorded. After euthanasia, the mandibles of the mice were examined using micro-CT.

**Table 1:** Comparison of bone mineral density indicators between clinical male patients taking olanzapine and healthy controls.

Male	Control	Olanzapine	*P*
Age (years)	22.79±2.83	22.88±3.81	0.9306
BMI	23.03±4.12	23.10±2.10	0.9477
Lumbar Spine BMD (g/cm^2^)	1.02±0.09	0.89±0.11	0.0001
Femoral Neck BMD (g/cm^2^)	0.96±0.12	0.77±0.10	0.0001
Hip BMD (g/cm^2^)	1.01±0.13	0.83±0.17	0.0004
Lumbar Spine Z-score	0.91±0.91	-0.36±1.19	0.0004
Femoral Neck Z-score	1.34±1.24	-0.49±1.03	0.0001
Hip Z-score	1.06±1.18	-0.09±0.95	0.0019

Data are presented as mean±standard deviation (Control Group N=24; Olanzapine Group N=17). Statistical significance was determined by unpaired t-test. *P*<0.05 was considered significant.

**Table 2:** Comparison of bone mineral density indicators between clinical female patients taking olanzapine and healthy controls.

Female	Control	Olanzapine	*P*
Age (years)	23.76±2.79	26.00±4.12	0.0928
BMI	21.07±2.98	22.55±1.46	0.1687
Lumbar Spine BMD (g/cm^2^)	0.98±0.09	0.91±0.09	0.0435
Femoral Neck BMD (g/cm^2^)	0.82±0.09	0.75±0.12	0.0738
Hip BMD (g/cm^2^)	0.89±0.08	0.85±0.09	0.1687
Lumbar Spine Z-score	0.32±0.93	-0.51±1.01	0.036
Femoral Neck Z-score	0.27±0.98	-0.63±1.26	0.0296
Hip Z-score	0.21±0.63	-0.30±0.96	0.092

Data are presented as mean ± standard deviation (Control Group N=21; Olanzapine Group N=9). Statistical significance was determined by unpaired t-test. P < 0.05 was considered significant.

After 12 weeks of olanzapine administration, mice in the experimental group weighed less than those in the control group (**Figure 1C**, **Supplementary Figure S1**). Further micro-CT results showed a decrease in bone mass between the first and second molars in the olanzapine group (**Figure 1D**), along with a significant reduction in bone volume fraction (BV/TV) and trabecular number (TbN), as well as a significant increase in trabecular separation (TbSp) (**Figure 1E**).

### 2.2 After olanzapine treatment, the composition of oral microflora in mice was changed

**Figure 2:**
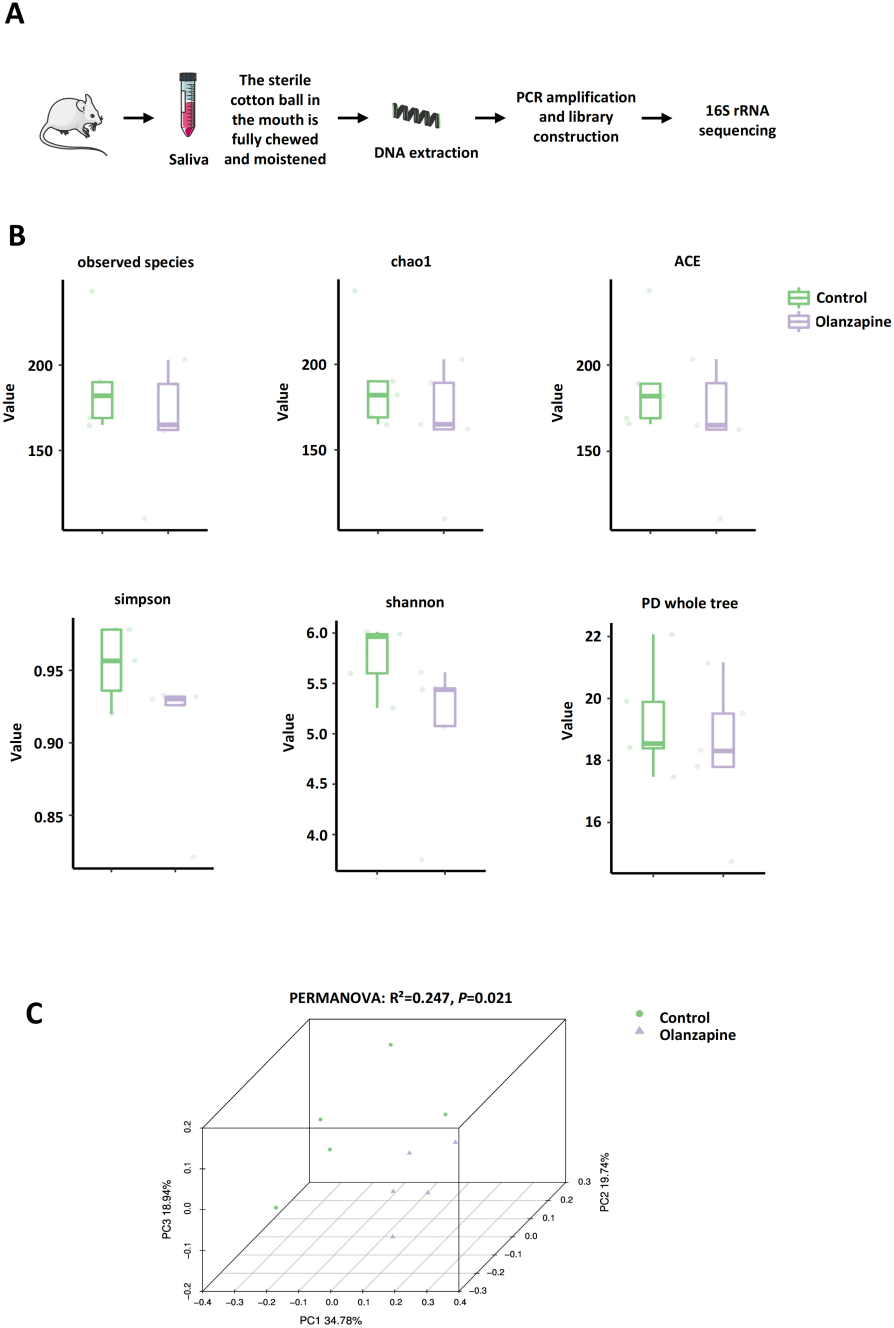
Effect of olanzapine on the oral microbiota. **(A)** Schematic diagram of saliva collection and 16S rRNA gene sequencing workflow. **(B)** α-Diversity analysis showing within-group microbial diversity, including observed species, Chao1, ACE, Simpson, Shannon, and PD whole tree indices. **(C)** Principal coordinate analysis (PCoA) was performed on weighted UniFrac distances to analyze differences in microbial community structure. PERMANOVA: R²=0.247, pseudo-F=2.62, *P*=0.021.

After olanzapine intervention, saliva from the mouths of mice was extracted for 16S rRNA sequencing (**Figure 2A**). The dilution curves of different samples eventually tended to be horizontal, indicating that the sequencing depth was sufficient and the sample sequencing volume was reasonable (**Supplementary Figure S2A**). The richness, evenness, and diversity of the oral microflora in mice were evaluated through α diversity analysis (**Figure 2B**). The results showed that after olanzapine intervention, the Observed Species index, Chao1 index, and ACE index, which represent the richness of the microbial community, showed no statistical differences. Additionally, the Simpson index, Shannon index, and PD whole tree index, which are closely related to the diversity of oral microbial communities in mice, also showed no statistical differences (**Figure 2B**).

Based on β diversity, 3D PCoA showed an apparent separation in oral microbial composition between control and olanzapine-treated groups (**Figure 2C**). Each point represents a sample, with closer distances indicating more similar communities. PERMANOVA (9, 999 permutations) confirmed a significant difference between groups (R²=0.247, pseudo-F=2.62, *P*=0.021).

### 2.3 Olanzapine may affect bone mass in mice by affecting the abundance of *Enterococcus*

**Figure 3:**
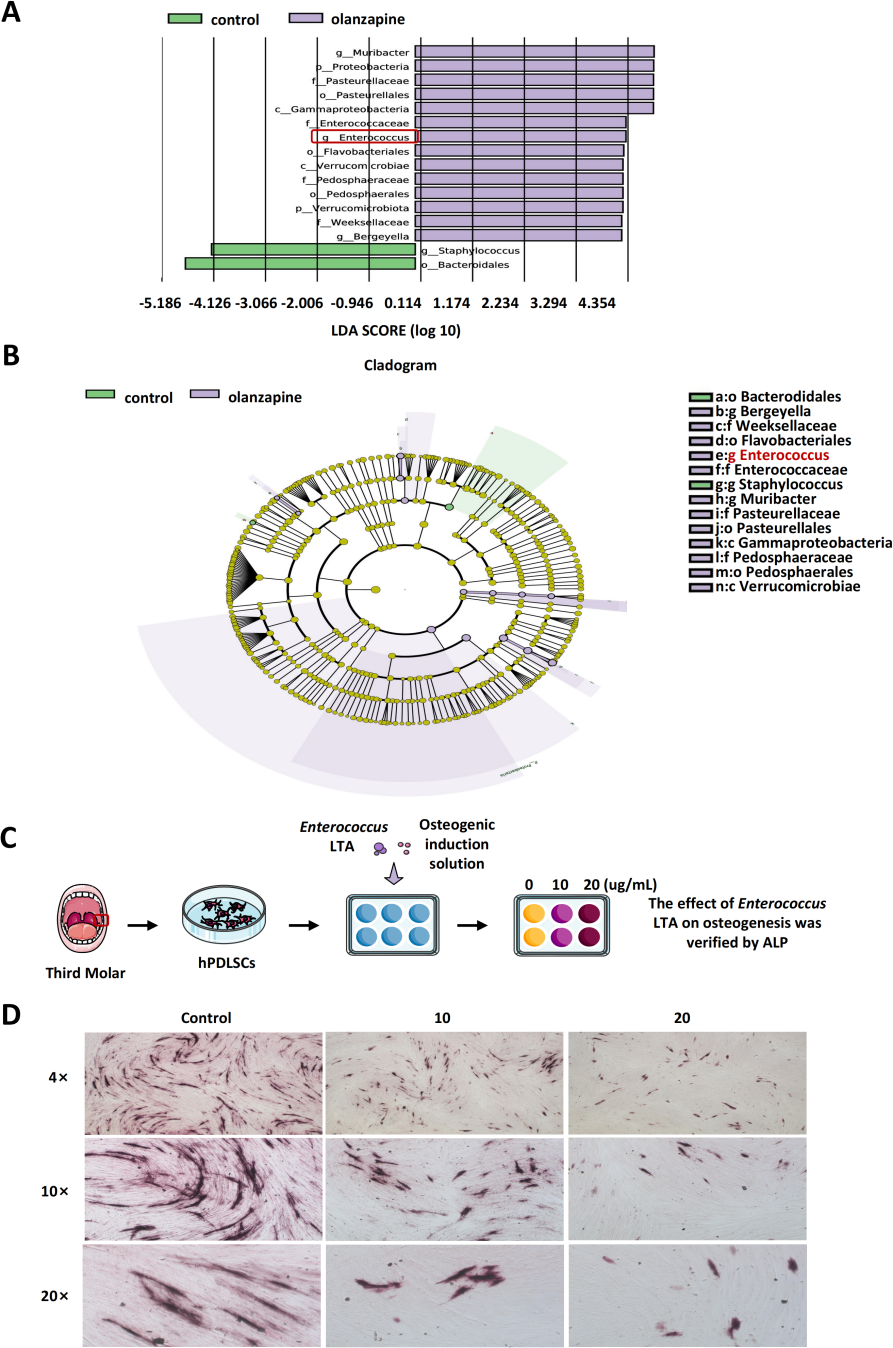
LEfSe analysis and alkaline phosphatase (ALP) staining. **(A)** LDA score bar plot showing microbial taxa with significant differences between control and olanzapine-treated groups, identified by LEfSe analysis. **(B)** Cladogram showing the phylogenetic relationships of differential taxa between the two groups. **(C)** Schematic diagram of human periodontal ligament stem cells (hPDLSCs) isolation from extracted third molars, followed by osteogenic induction with different concentrations of *Enterococcus* lipoteichoic acid (LTA) and ALP staining to assess osteogenic differentiation. **(D)** ALP staining results of hPDLSCs treated with *Enterococcus* LTA at concentrations of 0, 10, and 20 μg/mL, observed under 4×, 10×, and 20× magnifications.

Comparing the compositional differences of oral microbial communities in olanzapine-treated and non-treated mice, it was found that *Enterococcus*, *Muribacter*, and *Bergeyella* in olanzapine-treated mice were significantly increased at the genus level (| LDA score | ≥2, *P*<0.05). Meanwhile, *Staphylococcus* significantly decreased (**Figures 3A, B**; **Supplementary Figures S2B, C**). Among the significantly increased bacteria, *Enterococcus* was closely related to bone metabolism. As can be seen from **Supplementary Figure S2C**, there was almost no *Enterococcus* in the saliva of olanzapine-untreated mice, while the relative expression of *Enterococcus* in the oral cavity of olanzapine-treated mice ranged from 0.0001 to 0.0018. *Enterococcus* was among the top predictive genera identified by the random forest model (**Supplementary Figure S2D**). In a small ancillary pilot based on human saliva, the relative salivary *Enterococcus* load was higher in the olanzapine group (17.35±1.15) than in controls (1.00±0.19) (*P* < 0.05) (**Supplementary Figure S2E**).

After that, lipoteichoic acid (LTA) from *Enterococcus* was extracted. After 7 days of treatment with 0, 10, and 20 μg/mL LTA, alkaline phosphatase (ALP) staining showed a significant, concentration-dependent reduction in staining intensity in the LTA-treated hPDLSCs (**Figures 3C, D**). These results indicate that *Enterococcus* LTA has an inhibitory effect on the osteogenesis of hPDLSCs, and that the inhibitory effect is stronger with higher concentrations within a certain range.

In previous experiments, we found that olanzapine intervention led to a decrease in bone mass in both humans and mice. At the same time, after olanzapine intervention, *Enterococcus* appeared in the saliva of mice, which was not previously present. In parallel, its abundance also increased in the saliva of individuals taking olanzapine. Therefore, we suspect that *Enterococcus* may be the possible cause of olanzapine-induced bone mass change, but the relevant mechanism needs to be verified later.

### 2.4 After olanzapine treatment, the extracellular matrix of mouse bone tissue showed alterations

**Figure 4:**
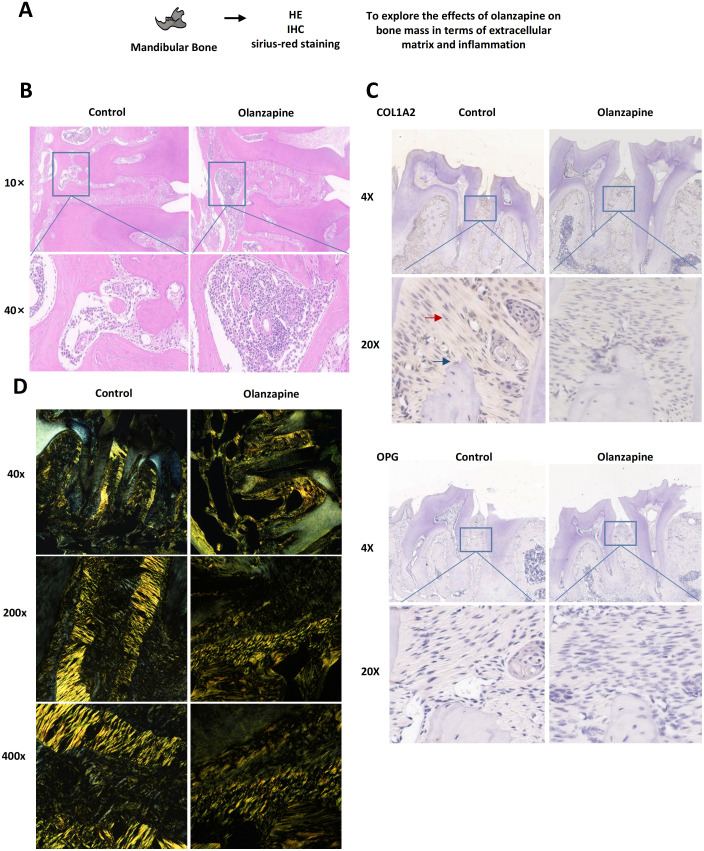
Effect of olanzapine on periodontal tissue in mice. **(A)** Schematic diagram illustrating the experimental design for mandibular bone staining to investigate the effects of olanzapine on bone mass via extracellular matrix and inflammation. **(B)** Hematoxylin and eosin (HE) staining for histological evaluation. **(C)** Immunohistochemical (IHC) staining for COL1A2 and OPG to assess collagen expression and osteogenic regulation. Red arrows indicate PDLSCs, and blue arrows indicate osteoblasts (OB). **(D)** Sirius red staining under polarized light microscopy to assess collagen fibers.

We explored a plausible mechanistic hypothesis by which olanzapine causes bone loss from two aspects: inflammation and collagen formation (**Figure 4A**). Regarding inflammation, we observed immune cell infiltration in the bone tissue of olanzapine-treated mice, indicating local inflammatory activity (**Figure 4B**).

Next, we investigated collagen formation. The immunohistochemical staining results of the COL1A2 antibody showed that the brown staining in the periodontal membrane of the molar area in the olanzapine group was lighter than that in the control group (**Figure 4C**), with the deepest brown color observed in the periodontal ligament stem cells (PDLSCs) and osteoblasts (OB). Immunohistochemical staining of the OPG antibody showed no significant difference in color between the control group and the olanzapine group. After staining with Sirius red, the dense red bunched fibers in the periodontal membrane of both the olanzapine and non-olanzapine groups were visible under the optical microscope (**Supplementary Figure S3**). Under polarized light, a large number of bright yellow type I collagen fibers could be seen in the periodontal membrane of mice in the control group, exhibiting a dense and orderly arrangement. In contrast, the yellow type I collagen in the periodontal membrane of mice treated with olanzapine was significantly reduced, darkened in color, and exhibited a more disordered arrangement (**Figure 4D**).

It is worth mentioning that the staining changes in periodontal ligament stem cells and osteoblasts were most pronounced in the immunohistochemical staining. Considering the representativeness of the oral cavity, periodontal ligament stem cells were selected as experimental cells for *in vitro* studies.

### 2.5 *Enterococcus* LTA induced extracellular matrix changes by up-regulating *SAA1* gene

**Figure 5:**
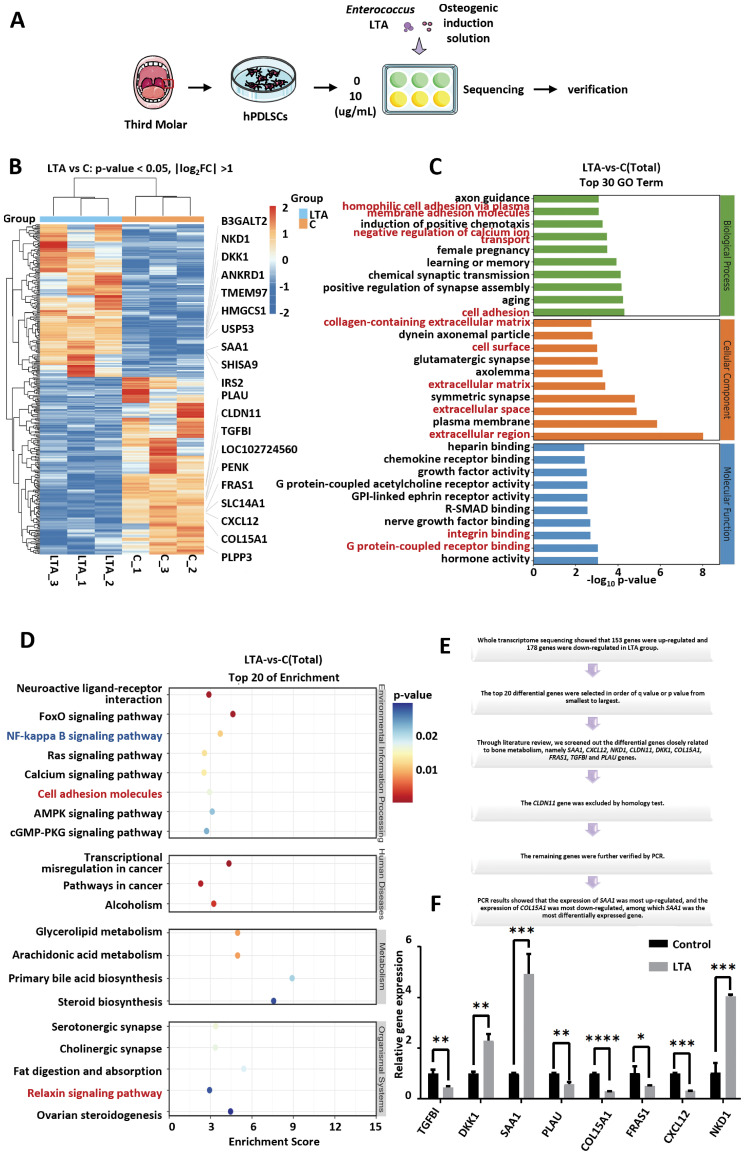
Whole-transcriptome sequencing and PCR validation of hPDLSCs after *Enterococcus* LTA treatment. **(A)** Schematic diagram of the experimental workflow for hPDLSCs isolated from third molars and treated with *Enterococcus* LTA, followed by transcriptome sequencing and PCR validation. **(B)** Heatmap of differentially expressed genes (DEGs) with |log_2_FC| > 1 and p-value<0.05 between the LTA-treated and control groups. **(C)** Gene Ontology (GO) enrichment analysis showing the top 30 enriched GO terms, categorized into biological process, cellular component, and molecular function. Extracellular matrix-related terms are labeled in red text. **(D)** Bubble plot of KEGG pathway enrichment analysis showing the top 20 enriched pathways. Pathways related to the extracellular matrix and inflammation are labeled in red and blue text, respectively. **(E)** Flowchart illustrating the screening process of candidate genes for further validation. **(F)** Relative mRNA expression levels of selected genes (*TGFBI, DKK1, SAA1, PLAU, COL15A1, FRAS1, CXCL12*, and *NKD1*) in hPDLSCs after *Enterococcus* LTA treatment, measured by PCR. Data are presented as mean±SD. Statistical analysis was performed using Student’s t-test. Significance levels: ns, not significant; *P*<0.05 (*); *P*<0.01 (**); *P*<0.001 (***); *P*<0.0001 (****).

To further investigate the mechanism, human periodontal ligament stem cells (hPDLSCs) were used to conduct an *in vitro* experiment. *Enterococcus* LTA was used to interfere with hPDLSCs for 7 days, and RNA was extracted for transcriptome sequencing (**Figure 5A**). The base quality distribution map predicts the probability of base discrimination errors. The average base error rate of each sample read at each site was found to be less than 0.04%, indicating that the sequencing quality evaluation was satisfactory (**Supplementary Figure S4A**). It was observed that there was relatively little difference within the same group, but significant differences between different groups (**Supplementary Figures S4B, C**). Compared with the control group, 153 genes in the hPDLSCs of the LTA group were up-regulated and 178 genes were down-regulated (**Supplementary Figure S4D**). The top 20 differentially expressed genes are shown in **Table 3** and **Figure 5B**. Among these, the expression of *SAA1*, *NKD1*, *USP53*, *DKK1*, *IRS2*, *SHISA9*, *TMEM97*, *B3GALT2*, *HMGCS1*, and *ANKRD1* genes was increased, while the expression of *CXCL12*, *CLDN11*, *SLC14A1*, *LOC102724560*, *COL15A1*, *FRAS1*, *PLPP3*, *PENK*, *PLAU*, and *TGFBI* genes was decreased. GO and KEGG enrichment analysis showed that the differentially expressed genes were closely related to extracellular matrix-related pathways and functions (**Figures 5C, D**).

**Table 3:** The top 20 differentially expressed genes (p-value<0.05, |log2FC| >1, ranked by q-value or p-value).

Gene id	Fold change	p-value	q-value	Regulation
SAA1	4.283057	3.81E-85	6.62E-81	Up
CXCL12	0.35498	5.57E-65	4.84E-61	Down
NKD1	3.600365	9.41E-53	5.45E-49	Up
CLDN11	0.327735	1.10E-49	4.76E-46	Down
SLC14A1	0.284362	1.87E-44	6.49E-41	Down
USP53	2.104107	3.89E-44	1.13E-40	Up
DKK1	2.172764	1.08E-43	2.68E-40	Up
IRS2	2.061607	1.30E-43	2.83E-40	Up
LOC102724560	0.023067	1.52E-38	2.94E-35	Down
SHISA9	3.661599	9.52E-37	1.65E-33	Up
COL15A1	0.389733	2.40E-36	3.79E-33	Down
FRAS1	0.404886	8.87E-33	1.19E-29	Down
TMEM97	2.45424	3.30E-31	4.09E-28	Up
B3GALT2	3.289264	8.74E-31	1.01E-27	Up
PLPP3	0.491811	1.16E-30	1.25E-27	Down
HMGCS1	2.177893	9.90E-30	1.01E-26	Up
PENK	0.452315	1.19E-28	1.15E-25	Down
ANKRD1	2.926935	8.55E-27	6.75E-24	Up
PLAU	0.478952	7.53E-26	5.69E-23	Down
TGFBI	0.435548	5.66E-24	4.10E-21	Down

Among the top 20 differentially expressed genes, *SAA1*, *CXCL12*, *NKD1*, *CLDN11*, *DKK1*, *COL15A1*, *FRAS1*, *TGFBI*, and *PLAU* genes were selected, as they were closely related to bone metabolism. The *CLDN11* gene was excluded based on a homology test, and the remaining genes were further validated by qPCR (**Figure 5E**). Additionally, most of the above gene-related pathways are also associated with the extracellular matrix or pathways with a high KEGG ranking, such as the NF-κB signaling pathway. The results of the qPCR showed that the expression of *SAA1* was most up-regulated, while the expression of *COL15A1* was most down-regulated (**Figure 5F**). *SAA1* was the most differentially expressed gene in the whole transcriptome sequencing. Therefore, *SAA1* was selected for further experiments.

### 2.6 *Enterococcus* LTA may inhibit the expression of *COL1A1* by promoting the expression of *SAA1*, and ultimately inhibit osteogenic differentiation

**Figure 6:**
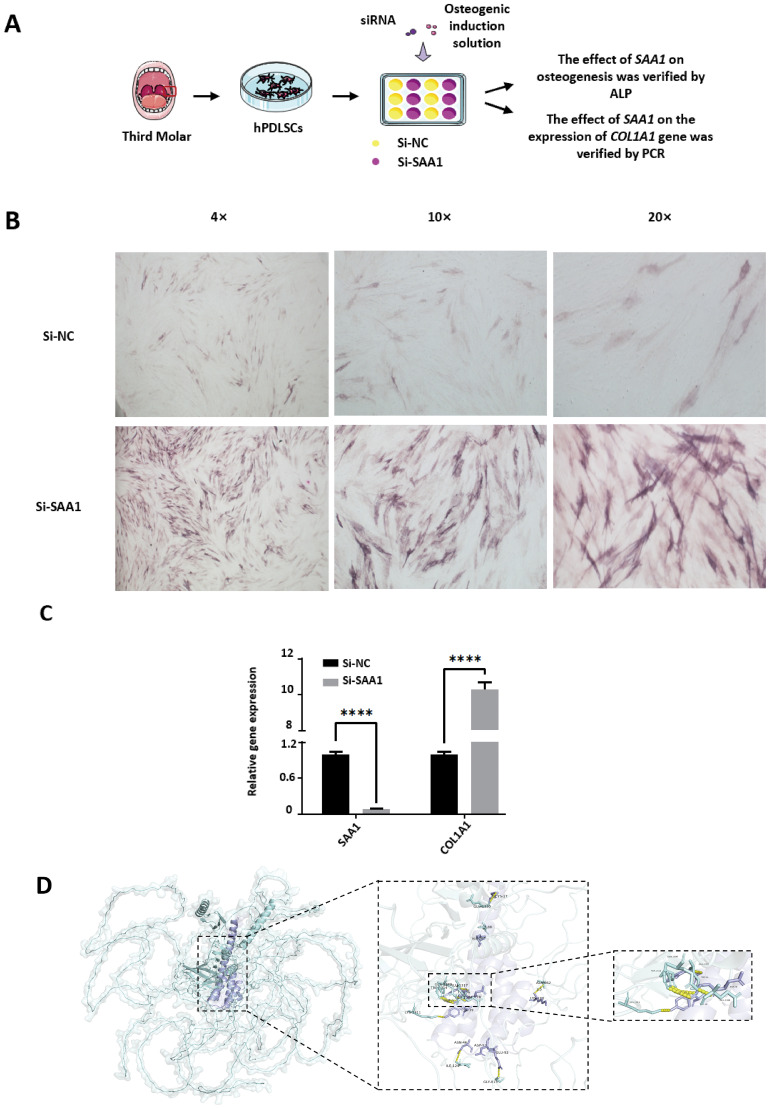
Effects of *SAA1* gene silencing on osteogenic differentiation and *COL1A1* expression in hPDLSCs, and predicted protein interaction between SAA1 and COL1A1. **(A)** Schematic diagram of the experimental workflow, including hPDLSCs isolation, siRNA transfection [Si-NC (negative control) or Si-SAA1 (SAA1 siRNA-transfected group)], osteogenic induction, ALP staining, and PCR analysis. **(B)** ALP staining of hPDLSCs after transfection with Si-NC or Si-SAA1, observed under 4×, 10×, and 20× magnifications. **(C)** Relative mRNA expression levels of *SAA1* and *COL1A1* in hPDLSCs after transfection with Si-NC or Si-SAA1, as determined by PCR analysis. Data are presented as mean±SD. **** indicates *P*<0.0001. **(D)** Predicted binding sites and interacting residues between SAA1 and COL1A1 proteins based on protein–protein interaction analysis.

In order to further explore the role of the *SAA1* gene in inhibiting osteogenic differentiation of *Enterococcus* LTA, the *SAA1* gene was inhibited in hPDLSCs, followed by ALP staining and qPCR assays (**Figure 6A**). In the ALP staining, inhibition of the *SAA1* gene in hPDLSCs enhanced the staining intensity and increased the stained area (**Figure 6B**). qPCR results indicated that after the inhibition of the *SAA1* gene, the expression of the *COL1A1* gene in hPDLSCs was up-regulated (**Figure 6C**). Inhibition of the *SAA1* gene led to the up-regulation of *COL1A1* gene expression, suggesting a possible interaction between the two. The relationship between SAA1 and COL1A1 was analyzed using PDBePISA website and PyMol tool, revealing that the two proteins were likely to bind and had multiple interaction sites (**Figure 6D**). The interface area between SAA1 (Structure 2) and COL1A1 (Structure 1) is 2689 Å^2^, and the free energy (ΔG) under this docking mode is -27.7 kcal/mol (**Supplementary Figure S5A**). **Supplementary Figure S5B** shows the number of atoms and residues, solvent-accessible area and solvation energy. All hydrogen bonds and salt bridges formed on the interaction surface are shown in **Supplementary Figure S5C**. Therefore, *Enterococcus* may inhibit COL1A1 at both the gene expression and protein action levels by promoting *SAA1* expression, thus inhibiting the osteogenesis of hPDLSCs.

## 3 Discussion

Through clinical trials and animal experiments, this study found that long-term olanzapine use can cause a reduction in bone mass, accompanied by an increase in *Enterococcus* abundance. Combined with cell experiments, this study suggests that these changes in *Enterococcus* are related to the *SAA1* gene. Based on this, a reasonable hypothesis can be made that olanzapine may increase the abundance of *Enterococcus*, and its component (LTA) may inhibit osteogenic differentiation through *SAA1*-mediated inflammatory and extracellular matrix–related signaling pathways, suggesting that *Enterococcus* may be involved in olanzapine-induced bone loss. More *in vivo* studies are needed to verify this in the future.

Olanzapine is commonly used to treat schizophrenia, but patients with schizophrenia who take olanzapine experience a decrease in bone mineral density, which is one of the common adverse effects of atypical antipsychotic drugs (Wyszogrodzka-Kucharska and Rabe-Jablonska, 2005). We found that olanzapine-treated mice also had lower bone mass and body weight than controls. Consistent with this, Langlais et al. (2023) and Li and Nagy (2013) used olanzapine to intervene in mice for 3–4 weeks and found that the trabecular bone of the femur in these mice was lost, with the volume and connectivity of the trabecular bone lower than that of the control group. The mechanism of weight change is complex and is related to factors such as gender, breed and environment. Previous studies have shown that olanzapine induces weight gain in female rats but has minimal effects on males, while some strains such as A/J mice even exhibit weight loss (Albaugh et al., 2006; Davey et al., 2012a).

However, the mechanism by which olanzapine causes abnormal bone metabolism remains unclear. Our 16S rRNA sequencing results showed that olanzapine could cause changes in oral microflora, but there was no significant difference in the diversity and richness of the microflora. Pelka Wysiecka et al. (2019) also found that after olanzapine treatment, there was no significant change in the α diversity of the microbiota of individuals with schizophrenia. However, Davey et al. found in a rat model that the diversity of the microbial community decreased (Davey et al., 2012b; Davey et al., 2013). This may be related to the strain of the animal and the way it is raised. We found that the abundance of *Enterococcus* increased after olanzapine intervention. We also found in the human samples that patients taking olanzapine had more *Enterococcus* in their saliva. *Enterococci* are Gram-positive bacteria that belong to the gastrointestinal flora of mammals (Hendrickx et al., 2009). Previous studies have shown that olanzapine has direct antibacterial effects on *Enterococcus* in the intestinal tract (Morgan et al., 2014). In the oral cavity, *Enterococcus* has been identified as a common root canal filling pathogen (Zehnder and Guggenheim, 2009). Intestinal flora may spread through the faecal-oral route and affect oral flora (Shaffer and Lozupone, 2018). In addition, diseases closely related to intestinal flora (such as inflammatory bowel disease and colitis) may also affect oral flora (Said et al., 2014; Rautava et al., 2015; Park et al., 2021), indicating that the intestinal flora may affect the oral flora through various ways. Olanzapine may further cause a compensatory increase of *Enterococcus* in the oral cavity after reducing *Enterococcus* in the intestinal tract. LTA is one of the main virulence factors of *Enterococcus* (Jett et al., 1994; Kayaoglu and Orstavik, 2004; Zehnder and Guggenheim, 2009). The literature indicates that *Enterococcus* is rare in the oral cavity of healthy individuals and is an opportunistic pathogen. When detected in the oral cavity, its abundance is often extremely low, yet it can exhibit significant pathogenicity and environmental tolerance (Zehnder and Guggenheim, 2009; Parga et al., 2025). This is consistent with our research. We did not detect *Enterococcus* in the saliva of the control group mice, while in the olanzapine group, *Enterococcus* was detectable but remained at a low abundance. However, even at such a low abundance level, *Enterococcus* could still cause significant local tissue damage (Zehnder and Guggenheim, 2009; Parga et al., 2025). We confirmed that *Enterococcus* LTA could inhibit osteogenic differentiation. Wang et al. (2016) used *Enterococcus* to interfere with osteoblasts *in vitro* and also found that osteogenic differentiation was inhibited. *In vivo* studies also indicate that *Enterococcus* is associated with reduced bone mass. For instance, *Enterococcus* infection has been proven to cause periapical inflammation in mice and to result in significant bone destruction (Dai et al., 2022). Beyond the oral cavity, Balish et al. discovered that *Enterococcus* can cause inflammatory bowel disease in mice (Balish and Warner, 2002), and such inflammation is closely related to systemic bone loss. In our research, the use of olanzapine occurred simultaneously with an increase in the abundance of *Enterococcus* in the oral cavity. Combined with the above evidence, this raises a hypothesis that *Enterococcus* may be one of the potential factors contributing to bone loss, but the mode of its influence still needs further exploration.

We found that *Enterococcus* LTA caused changes in the inflammatory pathway (NF-κB signaling pathway) of hPDLSCs. Consistent with this, the literature suggests that *Enterococcus* can destroy the epithelial barrier, promote intestinal inflammation (Steck et al., 2011), and may also be associated with different stages of root canal infection and periapical inflammation (Kayaoglu and Orstavik, 2004). We also found that the up-regulation of the *SAA1* gene was most significant in the whole transcriptome sequencing. The serum amyloid A1 gene (*SAA1*) encodes a member of the serum amyloid A apolipoprotein family. This is a major acute-phase protein highly expressed in response to inflammation and tissue damage. Previous studies have shown that microbial communities can alter the level of *SAA1* expression. After fecal microbiota transplantation, *Saa1* expression increased in mice (Huang et al., 2023). In mice treated with low protein microbiota (LPM) and high lipid microbiota (HFM), *Saa1* gene expression increased in the latter (Busnelli et al., 2020). Additionally, Mendelian randomization (MR) analysis found that increasing the abundance of Roseburia would decrease the level of SAA1 (Liu et al., 2024).

In addition, the extracellular matrix refers to the complex network of multiple macromolecules around the cell. Collagen is the main component of the extracellular matrix, in which type I collagen is composed of two α1 chains and one α2 chain. The extracellular matrix not only supports and connects, but also contains a large number of signaling molecules, which are closely related to osteogenesis. Our previous studies have shown that cytokine changes in extracellular matrix affected by microRNA can further affect the expression of collagen I α2 and cause osteogenic changes (Guo et al., 2016; Guo et al., 2017; Li et al., 2020). This time, we found a decrease in type I collagen in the extracellular matrix by bone tissue staining. Combined with the results of whole transcriptome sequencing, we hypothesized that extracellular matrix related pathway may be one of the main pathways that *Enterococcus* LTA inhibits osteogenic differentiation. Previous studies have also shown that *Enterococcus* can significantly down-regulate the expression level of collagen type 1 (COL1) (Wang et al., 2016), activate tissue matrix metalloproteinase 9 (MMP9) (Shogan et al., 2015), and degrade collagen and glycosaminoglycan (the main components of the extracellular matrix) (Shogan et al., 2015; Kawai et al., 2018).

We further inhibited the *SAA1* gene and found that *COL1A1* expression was up-regulated, enhancing osteogenic differentiation. Wang WS. et al. (2019) detected the expression level of *COL1A1* in amniotic fibroblasts, showing no significant difference. The reason for the difference with our results may be related to cell types and treatment methods. We also predict that the proteins encoded by *SAA1* and *COL1A1* may be bound by hydrogen bonds and salt bridges. ΔiG is less than zero, indicating that the docking results are meaningful. Specifically, amino acid 17–108 in SAA1 may interact with amino acid 88–1384 in COL1A1. Our molecular docking experiments show that residues such as Glu92, Asp78, and Gln98 in SAA1 and Gly183, Gln1380, and Glu1384 in COL1A1 may be important binding sites. Meanwhile, SAA1 has been shown to increase the abundance of collagenases MMP-1, MMP-8, and MMP-13 while decreasing the abundance of lysine oxidase-like 1, an enzyme that cross-links collagen (Han et al., 2016; Wang YW. et al., 2019; Lin et al., 2023). At the same time, Wang WS. et al. (2019) suggested that SAA1 might degrade type I collagen through autophagy and MMP pathways. Therefore, *SAA1* may inhibit osteogenic differentiation through extracellular matrix-related pathways by influencing the expression of the *COL1A1* gene, directly acting on *COL1A1*-encoded proteins, or indirectly affecting type I collagen through autophagy or enzyme-related pathways.

This study has some limitations. First, the *in vivo* model did not fully rule out other potential confounding factors induced by olanzapine, such as alterations in prolactin levels or metabolic pathways, which may also influence bone metabolism. Second, the relatively small sample size may limit the generalizability and statistical power of the findings. As such, the roles of *Enterococcus* and SAA1 should be interpreted as exploratory signals rather than established mechanisms. Future research should increase the sample size, adopt standardized animal models, and, where feasible, employ colonization or clearance models to directly assess the effects of *Enterococcus* on bone phenotypes.

In conclusion, we propose a plausible hypothesis that olanzapine may increase the relative abundance of oral *Enterococcus*, promote the expression of the *SAA1* gene, and thus inhibit the expression of the *COL1A1* gene. It may also affect the protein interaction between amino acids 17–108 in SAA1 and amino acids 88–1384 in COL1A1, ultimately inhibiting bone formation. These findings suggest a possible link between oral microbiota alterations and olanzapine-induced bone metabolism abnormalities, supporting further investigation into microbiota-informed strategies for reducing skeletal complications and maintaining oral health.

## 4 Materials and methods

### 4.1 Experimental design

The purpose of this study was to investigate the mechanism of olanzapine aggravating abnormal bone metabolism and causing bacterial imbalance in patients with schizophrenia. This study verified the effect of olanzapine on bone metabolism through clinical trials. A mouse model of olanzapine administration was established to investigate the changes of bone tissue and oral flora. The effect and mechanism of related bacteria on osteogenesis were further explored by cell experiment and prediction of protein interaction.

### 4.2 Clinical trials

A total of 71 participants were enrolled in this study. The olanzapine group consisted of 26 patients (17 males and 9 females), while the healthy control group included 45 individuals (24 males and 21 females), with age-matching performed between the two groups. Inclusion Criteria: Patients in the olanzapine group met the diagnostic criteria for schizophrenia according to the Diagnostic and Statistical Manual of Mental Disorders, Fourth Edition (DSM-IV), and their diagnosis was independently confirmed by two or more psychiatrists with attending or senior titles. Eligible patients were between 20 and 35 years old (inclusive), received monotherapy with olanzapine at doses ranging from 5 to 25 mg/day, and had been on continuous and regular olanzapine treatment for more than 6 months. The healthy control group consisted of adults aged between 20 and 40 years with no history of psychiatric illness. Exclusion Criteria: Participants were excluded if they had taken medications affecting bone metabolism for more than 6 months, such as bisphosphonates, glucocorticoids, parathyroid hormone, calcitonin, or active vitamin D analogs. Other exclusion criteria included diseases that may affect bone metabolism, such as uncontrolled diabetes mellitus (HbA1c > 7%), hyperthyroidism or hypothyroidism, primary parathyroid disorders, osteoarthritis, unresolved skeletal disorders, or a history of radiation therapy or chemotherapy within the past 6 months. Subjects with contraindications for dual-energy X-ray absorptiometry (DXA) were also excluded.

This study protocol was approved by the Human Research and Ethics Committee of the Second Xiangya Hospital of Central South University. The clinical study was registered on the ClinicalTrials.gov platform of the U.S. National Institutes of Health (Registration Number: NCT06123897). All procedures strictly adhered to the ethical guidelines outlined in the Declaration of Helsinki. All experiments were conducted in accordance with relevant guidelines and regulations. Written informed consent was obtained from all participants after a thorough explanation of the study procedures. All procedures associated with this study were performed strictly after obtaining written informed consent from each participant.

Bone mineral density (BMD) of the lumbar spine, femoral neck, and hip bone was measured using a dual-energy X-ray absorptiometry (DEXA) scanner (Hologic, USA). Given the significant gender- and age-dependent variation in physiological bone density, Z-scores were calculated to minimize related influences. The Z-score was defined as follows: Z-score=(Measured BMD - Mean BMD of age-, sex-, and ethnicity-matched reference population)/Standard deviation of BMD in the reference population.

An ancillary saliva pilot (n=3) was conducted. Exclusions: antibiotics within 3 months; antiseptic mouthwash on the sampling day; dental/periodontal procedures within 3 months; active oral infection or acute respiratory illness; current smoking; major systemic disease or immunosuppression. Unstimulated whole saliva was collected by passive drooling after ≥30-min abstinence from eating, drinking, or oral hygiene, immediately aliquoted, and stored at −80 °C.

### 4.3 Olanzapine animal modeling

3-month-old male C57BL/6J mice (Hunan Slake Jingda Laboratory Animal Co., LTD., China) weighing 20-24g were selected. The mice were quarantined and acclimatized for 1 week, then randomly assigned to different treatment groups using a computer-generated randomization schedule. Weight before administration<18 or > 26 g or mice in poor physical condition were excluded from the experiments. All mice were maintained under specific pathogen-free conditions in an animal facility with controlled temperature (22 ± 2 °C), humidity (50 ± 10%), and a 12-hour light/dark cycle. Mice had ad libitum access to autoclaved standard chow and sterile water. The mice were divided into olanzapine and control groups, with 10 mice in each group. Olanzapine group: 3-month-old mice were treated with olanzapine (4 mg/kg/day) by gavage for 12 weeks. Mice were given solvent using the same treatment protocol served as healthy controls. Group allocation and outcome assessment were performed in a blinded manner to reduce bias. Changes in body weight were recorded weekly. The dose of olanzapine was adjusted for each mouse. All animals were monitored daily for signs of distress, and predefined humane endpoint were established but not triggered during the study.

The animal study was reviewed and approved by the Experimental Animal Ethics Committee of the Second Xiangya Hospital. All experiments were performed in accordance with relevant named guidelines and regulations. The authors complied with the ARRIVE guidelines.

### 4.4 16S RNA identification

(1) Sample extraction: Take a sterile cotton ball, place it in the mouse’s mouth, allow it to be fully chewed, then remove the fully wetted cotton ball and place it into an EP tube. Preserve it at -80 °C, transport it in liquid nitrogen, and send it to Shanghai Ouyi Biotechnology Co., Ltd. for 16S rRNA sequencing.

(2) DNA extraction: We use MagPure Soil DNA LQ Kit based on the instructions provided by the reagent vendor (Magen China). The kit separates DNA from each sample. Using the NanoDrop 2000 Spectrophotometer (Thermo Fisher Scientific, MA, USA) and Agarose Gel Electrophoresis (Depending on the situation) DNA yield, purity, and integrity were assessed.

(3) PCR Augmentation and Library Construction: Use universal primer 343F: 5'-TACGGRAGGCAGCAG-3' and 798R: 5'-AGGGTATTAATCCT-3') PCR amplification was performed on V3-V4 high-variation regions of 16S rRNA gene. Both primers are connected to the Illumina sequencing adapter. Gel electrophoresis is used to visualize the quality of amplified products. The Agencourt AMPure XP bead (Beckman Coulter Co., USA) was used to purify PCR products and quantify them with Qubit dsDNA assay kits. The concentration is then adjusted for sequencing.

(4) High throughput sequencing, bioinformatics: Sequencing is using EE (OE Biotech Shanghai, China) the Illumina NovaSeq6000 high-throughput sequencing platform, Follows its standard procedures. The original data is in FASTQ format, and after the data is disembarked, the pair of end readings are pre-processed using cutadapt software to detect and disconnect the adapter. After trimming, use DADA2 and QIME2 (2020.11) The default parameter filters low-quality sequences, de-noises, merges, and detects and cuts chimeric readings for pairing end readings to obtain representative sequences and ASV abundance tables. Use the QIIME 2 package to select the representative sequences for each ASV and compare all the representative sequences with the database. 16S uses the Silva (version138) database comparison. Species comparison annotations are analyzed using the q2 feature classifier default parameters Statistical analysis of microbial composition and function was carried out at species and genus levels. The differential expression of bacterial genes was identified by comparing relative abundance. The Wilcoxon rank and test are analyzed to assess the differences in the relative abundance of microbial communities. Bacterial abundance and diversity among samples were assessed using the α index (ACE, Chao1, Shannon, and Simpson). Based on relative abundance, the t-test is used to compare the α diversity index. We use Primary Coordinate Analysis (PCoA) to map similarities or differences in sample community composition. In addition, a similarity analysis (Adonis) was conducted to assess the significance of the differences in community structure between the two groups using the β diversity index. Use the default parameter (α parameter with a significant threshold set to 0.05 and the logarithmic LDA score cut-off set to 2.0) to perform a differential taxon analysis using the linear discriminant analysis effect size (LEfSe). We used the random forest model as an exploratory predictive method to assess the contribution of *Enterococcus* to group discrimination.

### 4.5 μCT analysis

After 12 weeks of continuous oral gavage with olanzapine (4 mg/kg/day), the mandibular alveolar bone was collected and placed in polyformaldehyde for two days. Following this, the alveolar bone was rinsed with tap water 10–12 times, dehydrated in 50% alcohol for 30 minutes, and then dehydrated in 70% alcohol for another 30 minutes at 4 °C.

Treated mandibles from all experimental groups were analyzed with micro-computed tomography (micro-CT) (Aoyin AX2000ct, Shanghai, China). X-ray imaging was conducted at a tube voltage of 90 kV and a tube current of 80 μA, with an image acquisition time of 2 minutes and a pixel size of 9.5 μm/100 μm. VGstudio MAX 3.4.4 (Volume Graphics, Inc., Heidelberg, Germany)was used to analyze the bone mass between the roots of the first and second molars in mice.

### 4.6 HE

(1) Burying and Slices

Fresh mouse mandible tissue was immediately placed in polyformaldehyde fixation solution for more than 24h, followed by EDTA decalcification for 28 days, Changing the solution every 7 days, The tissue was then trimmed and places into a embedding box for dehydration and infiltration with wax for paraffin embedding.

Finally, the wax block was placed in the slicer, with the mouse molar’s oriented from near to far as the axis, and cut into 4 μm slices. The slices were baked in an oven at 60°C. After drying, the wax blocks were removed and stored at room temperature.

(2) HE

a) Dewaxing and Hydration: Paraffin slices are dewaxed in xylene solution for 20 minutes, followed by 5 minutes in a gradient ethanol solution, and then placed in distilled water for 3 minutes. b) Hematoxylin staining: The slices are stained with hematoxylin solution for about 3–5 minutes, washed with tap water for about 10 minutes, treated with 1% hydrochloric acid differentiation solution for about 3 seconds, and finally placed in flowing water to restore the blue color. c) Eosin staining: The slices are stained in 0.5% eosin Y solution 5min. d) Dehydration and Sealing: Slices are placed in gradient ethanol and xylene solution 2min, then sealed with neutral gum.

### 4.7 IHC

The slides were placed in an oven at 65 °C for 4–6 hours, followed by dewaxing with turpentine, gradient ethanol, distilled water, and PBS. Next, the tissue was repaired with 0.25% trypsin. After cooling to room temperature, a circle was drawn around the tissue with an immunohistochemical pen at a distance of about 2 mm. The slides were incubated with 3% H_2_O_2_ to avoid light exposure. Afterward, 50 μL of anti-Collagen I alpha2 (Affinity USA) and OPG (Abcam USA) were added to each slide, and they were stored at 4 °C overnight. For the negative control, PBS or antibody diluent was added instead.

After overnight incubation, bring to room temperature, discard the antibody fluid, rinse with PBS, add 50–100 μL of HRP-marked goat anti-rabbit IgG (Servicebio China), incubate at room temperature for 30–60 min. Following this, 50-100μL DAB working solution (Servicebio China)was applied to each slide. Distilled water was used to terminate color reaction, followed by counterstaining with hematoxylin. The slides were rinsed with tap water to restore the blue color, subjected to gradient dehydration (80%, 95%, 100%), sealed with neutral gum, and observed under a dry lens.

### 4.8 Sirius red staining

The slices were put into turpentine, gradient ethanol, distilled water, and PBS for dewaxing. Slices were stained with Sirius red dye for 8min, dehydrated with anhydrous ethanol; After that, the slices were placed in a clean xylene solution for 5min, and sealed with neutral gum. Microscope examination, and image acquisition were performed for analysis.

Results: Collagen fiber appear red under an optical microscope, with a yellow background. In polarized light, type I collagen appears as orange or bright red coarse fiber, and type III collagen appears as green fine fiber.

### 4.9 Acquisition and culture of PDLSCs

(1) Enzyme preparation:

60mg of type I collagenase (Abiowell China)was placed in a 15mL centrifugal tube; 80mg of dispase (SIGMA-ALDRICH USA)was placed in a 15ml centrifugal tube, dissolved in 10 mL PBS with 1% P/S, and filtered, stored at 4°C.

(2) PDLSCs extraction:

- After extraction(it is best to harvest cells as soon as possible, within 4 hours extraction, need to be transported with an ice box), add the free teeth directly into the 15ml centrifugal tube containing PBS with 1% P/S, and stored at 4°C.
- Place the extracted teeth upside down on the dish (Root up) in the clean bench, rinse the root from top to bottom with PBS containing 1% penicillin/streptomycin to remove blood and other substances until rinsed clean, put teeth into another dish;
- Use a sterile scalpel to scrape the periodontal ligament from the middle third of the root, rinse 3 times with 1% penicillin/streptomycin-PBS, transfer it to another dish, and cut it into pieces;
- Add 1ml each of type I collagenase and dispase, seal the tube, and digest 45-60min in 37° incubator, shaking every 10–15 minutes, Until there is no distinct tissue blocks or flakes, transfer the mixture into a 15ml centrifugal tube, centrifuge at 1100rpm, 7min.
- Remove the upper layer, add 5mL conditioned medium, which consisted of αMEM (Gibco USA) containing 10% FBS (Gibco USA) and 1% penicillin/streptomycin (Gibco USA), transfer the mixture to a T25 flask, and change the medium every 3–4 days.
- The attached cells can be seen under the microscope and should reach 70–80% confluency before passaging.

(3) Cultivation of PDLSC: change the medium every 3–4 days with conditioned medium.

### 4.10 LTA intervention

*Enterococcus* hirae LTA (Sigma-Aldrich, USA), is dissolved in a phosphate buffer to a concentration of 5 mg/mL as the stock solution and sterilized using a 0.22μm filter. Before application, the LTA solution is diluted with a conditioned medium to the desired concentration. All solutions are prepared at room temperature and pH is adjusted to 7.4.

Conditioned Medium Configuration: The conditioned medium consists of 10% bovine serum, 1% penicillin/streptomycin in α-MEM.10 and 20 μg/ml *Enterococcus* LTA is applied to hPDLSCs cells in a pore plate and induces osteogenesis for 7 days, with the medium changed every 3 days.

### 4.11 ALP

The cells were plated in six-well plates and treated with 0 μg/mL, 10 μg/mL and 20 μg/mL of *Enterococcus* LTA osteogenic induction medium, which contains αMEM, 10% FBS, 100 μg/mL ascorbic acid, 10 mM β-glycerophosphate, dexamethasone, and 1% penicillin/streptomycin. The medium containing LTA was replaced every 3 days. After 7 days of intervention, the fixation solution was prepared in a 9:1 ratio of methanol to formaldehyde. The 0.2g of pentobarbital sodium, 0.2g of calcium chloride, 0.3g of β-glycerol, and 0.4g of magnesium sulfate were dissolved in 10mL of distilled water. The solution was then incubated and stored at 4°C. After washing cell-cultured plates with PBS, the fixation solution was added for 30 seconds, followed by a quick rinse with PBS. Fresh incubation fluid a then added, and the plates were incubated at 37°C for 4 hours. The fluids were removed, and the plates were rinsed before soaking in a 2% cobalt nitrate solution for 5 minutes. After rinsing, 1.25% ammonium sulfide was added for 10 seconds, followed by another rinse. Finally, the cells were stained with 2% sand yellow dye for 10 seconds, rinsed, and dried. Microscopically, a positive reaction was indicated by a gray-black granular or flaky precipitate located in the cytoplasm.

### 4.12 Total RNA sequencing

Sample collection: For the *Enterococcus* LTA 10 μg/mL intervention and osteogenesis after 7 days of hPDLSCs treatment, add 1 mL of TRIzol per 10 cm² of cells. Then move the frozen storage tube into -80 °C standby.

RNA extraction and library construction: Total RNA was extracted using TRIzol reagents according to the specification. The purity of the RNA was identified using the NanoDrop 2000 Spectrophotometer (Thermo Scientific, USA), and RNA integrity was assessed using the Agilent 2100 Bioanalyzer (Agilent Technologies, Santa Clara, CA, USA). The VAHTS Universal V5 RNA-seq Library Prep kit was used to construct a transcriptome library based on the instructions. Transcriptome sequencing and analysis are carried out in Shanghai, China.

RNA Sequencing and Differential Expression Gene Analysis: the library was sequenced using the Illumina NovaSeq 6000 sequencing platform generating 150bp paired-end reads. Fastp software were used to process the raw reads in FASTQ format, removing low-quality reads to obtain clean reads for subsequent data analysis. HISAT2 software was employed to align the reads to the reference genomes, calculate gene expression levels (FPKM), and obtain gene counts using HTSeq-count. Principal Component Analysis (PCA) was conducted using R (v 3.2.0) to assess biological duplication of the samples. Differential expression gene analysis was performed using DESeq2 software, with genes defined as differentially expressed (DEGs) if they met the thresholds of q<0.05 and fold change > 2 or fold change<0.5. Hierarchical clustering of DEGs was carried out using R (v 3.2.0) to demonstrate gene expression patterns across different groups and samples. An R package was used to visualize the top 30 genes, showing changes in the expression of upregulated and downregulated genes. Subsequently, Gene Ontology (GO), KEGG Pathway enrichment analyses were performed based on hypergeometric distribution algorithms to filter significant enrichment entries. R (v 3.2.0) was used to create column diagrams, or enrichment analysis circles for significant enrichment feature entries.

### 4.13 qPCR

The Quick RNA Extraction Kit (Accurate Bio-Medical Hunan, China) is utilized to extract mRNA from the PDLSCs. Next, 1 μg mRNA of satisfactory quality is selected for reverse transcription to obtain cDNA (reverse transcription kit Cat# AG11728 Accurate Bio-Medical Hunan, China), which is subsequently subjected to qPCR (SYBR green qPCR kit Cat#AG11701Accurate Bio-Medical Hunan, China). RocheLightCycler^®^480, The PCR cycle conditions are 95 °C, 15min, followed by 40 two-step cycles at 94 °C, 10s and 60°C, 45s, using a comparison cycle threshold (Cycle threshold, Ct). The primer sequences are provided in **Table 4**. The relative mRNA abundance of the target genes was normalized to β-actin and was then calculated using the 2-ΔΔCt method.

**Table 4:** Nucleotide sequences of primers used for qPCR.

Target	Direction	Primers (5′→3′)
SAA1	Forward	CAGACAAATACTTCCATGCTCG
	Reverse	TCTCTGGATATTCCTCTGGCA
TGFBI	Forward	ACTCAGCCAAGACACTATTTGA
	Reverse	CTTGTATGGGCATCAATTGGAG
PLAU	Forward	AATTTCAGTGTGGCCAAAAGAC
	Reverse	GTCCTCCTTCTTTGGGTAATCA
FRAS1	Forward	CCAAGTATACGAGCATGGTGAG
	Reverse	TACCGCACAACTCCATCATAG
COL15A1	Forward	GATCATCCTCTACTACACGGAG
	Reverse	CTTCCTCACCCTGGACAATCAT
DKK1	Forward	TACCAGACCATTGACAACTACC
	Reverse	TCCATTTTTGCAGTATTCCCG
NKD1	Forward	ACCATTGCGTAGATGAGAACAT
	Reverse	AAATTGGGACGTGTAGTTTT
CXCL12	Forward	CCAACGTCAAGCATTCAAAAT
	Reverse	CACACTTGTCTGTTGTTGTTCT
COL1A1	Forward	AGGCGAACAGGGCGACAGAG
	Reverse	GGACCAACAGGACCAGCATCAC
ALPL	Forward	ACCGCACGGAACTCCTGACC
	Reverse	TGCCTGCCTGCCCGATGG
BGLAP	Forward	GCCAGGCAGGTGCGAAGC
	Reverse	GCCGATGTGGTCAGCCAACTC
RUNX2	Forward	AGCAGCAGCAGCAGCAACAG
	Reverse	GCAGCACCGAGCACAGGAAG

The HiPure Bacterial DNA Kit (Magen, China) was utilized to extract DNA from human saliva. 100 ng of DNA was subsequently subjected to qPCR (2×HS Taq Universal SYBR Green qPCR Master Mix, Richamp, Hunan, China). The PCR was performed on a CFX Opus 96 Real-Time PCR System (Bio-Rad, USA) under the following cycling conditions: 95°C for 30 s, followed by 40 three-step cycles at 95°C for 10 s, 50°C for 10 s, and 72 °C for 30 s, using the comparative cycle threshold (Ct) method. SYBR Green qPCR quantified salivary *Enterococcus* using 23S rRNA primers ECST748F (5′→3′: GAGAAATTCCAAACGAACTTG) and ENC854R (5′→3′: CAGTGCTCTACCTCCATCATT); total bacterial load for normalization was measured using universal 16S rRNA primers EUB338 (5′→3′: ACTCCTACGGGAGGCAGCAG) and EUB518 (5′→3′: ATTACCGCGGCTGCTGG), and relative abundance was calculated using the 2^−ΔΔCt method.

### 4.14 Transfection

Cell transfection: The 4th to 6th generations of hPDLSCs were inoculated in 12-well plates and divided into 3 groups: positive control group, negative control group, and knockdown group. Transfection was performed when the cell confluence reached 60-80%. siRNA was purchased from Guangzhou Ribobio Co., Ltd. (Guangzhou, China). According to the instructions, Lipofectamine™ 2000 was used to transfect the three groups of hPDLSCs.

### 4.15 Protein interaction

Protein PDB files for SAA1 and COL1A1 were downloaded from the Uniprot database (P05366·SAA1_MOUSE; P11087·COL1A1_MOUSE). Predictions of potential interaction interfaces between SAA1 and COL1A1 were obtained from the GRAMM website. Then the data of binding energy, hydrogen bond and salt bridge were predicted by PDBePISA website. Finally, PyMol tool (https://pymol.org) was used to visualize the prediction results.

### 4.16 Statistical analysis

Data are the mean±standard deviation. Statistical analyses were carried out using one-way analysis of variance. A comparison of two groups was assessed by Tukey’s *post-hoc* comparisons. Some samples are two groups of data, and unpaired t-test is used. *P <*0.05 was considered significant.

For clinical research, based on exclusion criteria, prior literature and *a priori* causal framework, we considered the following potential confounders: age, sex and BMI. Continuous covariates were mean-centered; categorical covariates were represented with indicator variables. We used linear regression (separate models for each skeletal site). We report β coefficients as mean differences in Z-score for olanzapine users versus non-users with 95% confidence intervals. Two-sided *P* values<0.05 were considered statistically significant. Analyses were performed in R (version 4.4.0).

## Data Availability

The data presented in the study are deposited in the NCBI Sequence Read Archive (SRA) under accession numbers PRJNA1370146 and PRJNA1370464, and in the Gene Expression Omnibus (GEO) under accession number GSE311844.
