# Supplementary material for: Olanzapine affects bone formation via oral Enterococcus through SAA1 gene and extracellular matrix-related pathways
Source: Front Cell Infect Microbiol. 2026 Jan 6;15:1673931. doi: 10.3389/fcimb.2025.1673931 (PMC12815755; doi:10.3389/fcimb.2025.1673931)
Supplement: Supplementary file 1 [file SupplementaryFile1.docx]

Supplementary Material

# Supplementary Figures





**Weight (g)**

**Time (d)**

**A**

**B**

**Control**

**Olanzapine**


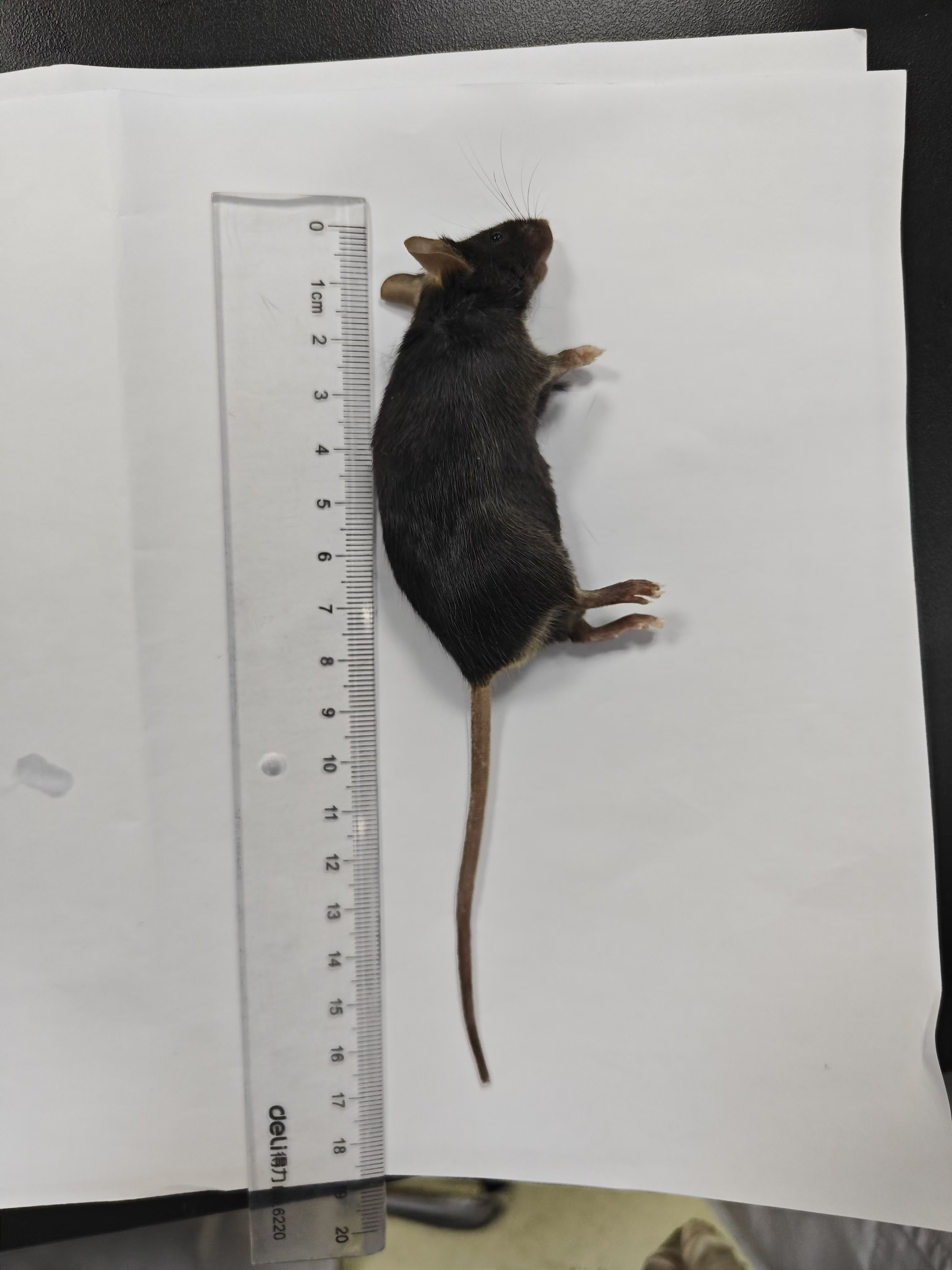

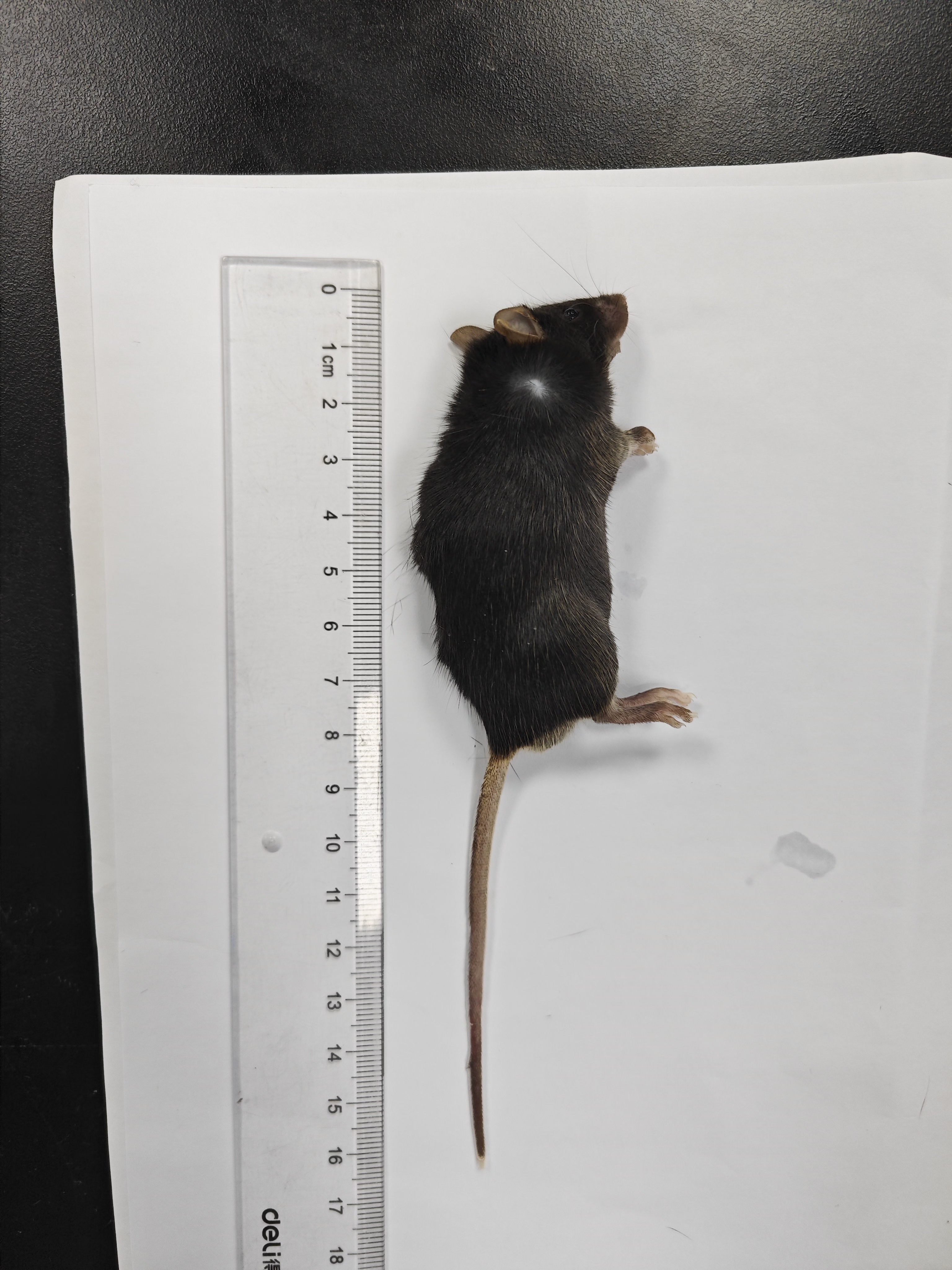


**Supplementary Figure 1.** **Body weight monitoring and gross appearance of mice in the olanzapine-treated and control groups.**
(**A**) Body weight changes of individual mice. Mice labeled with “C” represent the control group, and those labeled with “O” represent the olanzapine-treated group.
(**B**) Representative gross appearance of mice.


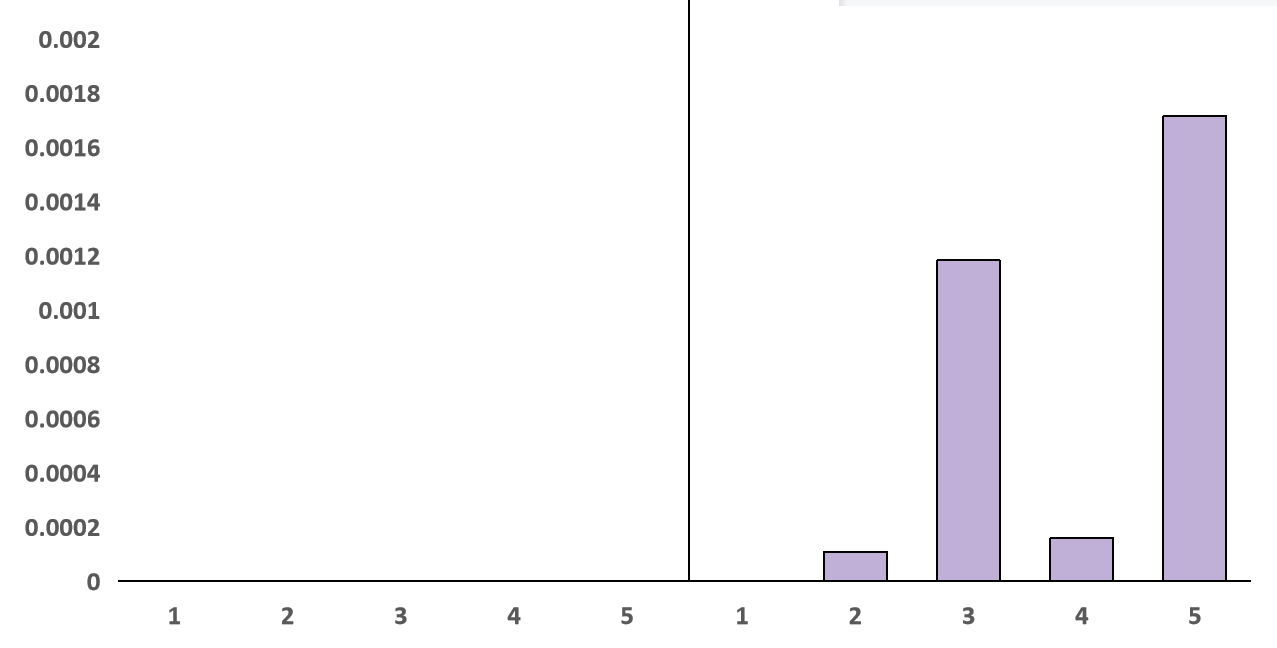


**olanzapine**

**control**

**Relative abundance**

**k Bacteria.p Firmicutes.c Bacilli.o Lactobacillales.f Enterococcaceae.g Enterococcus**

**C**


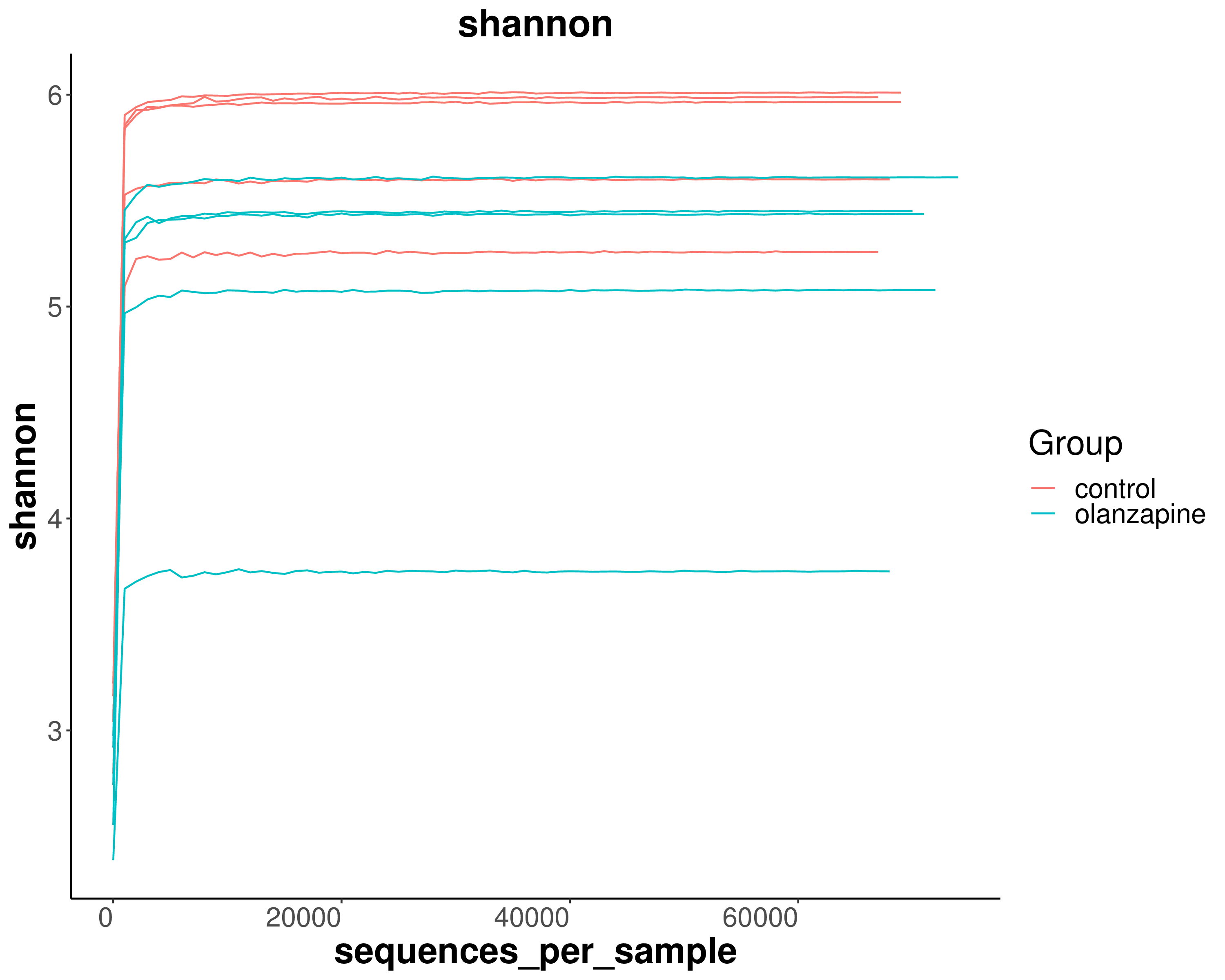

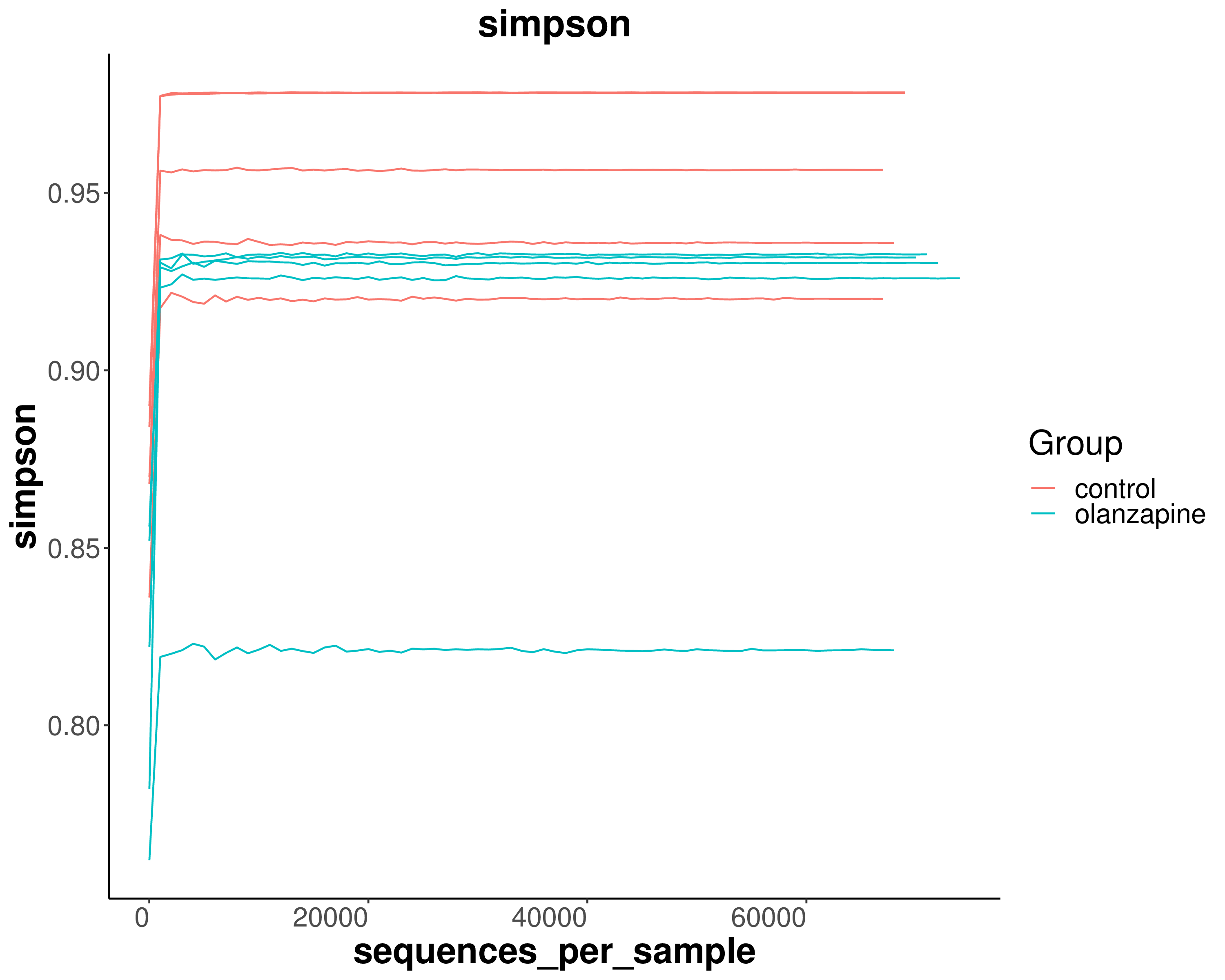

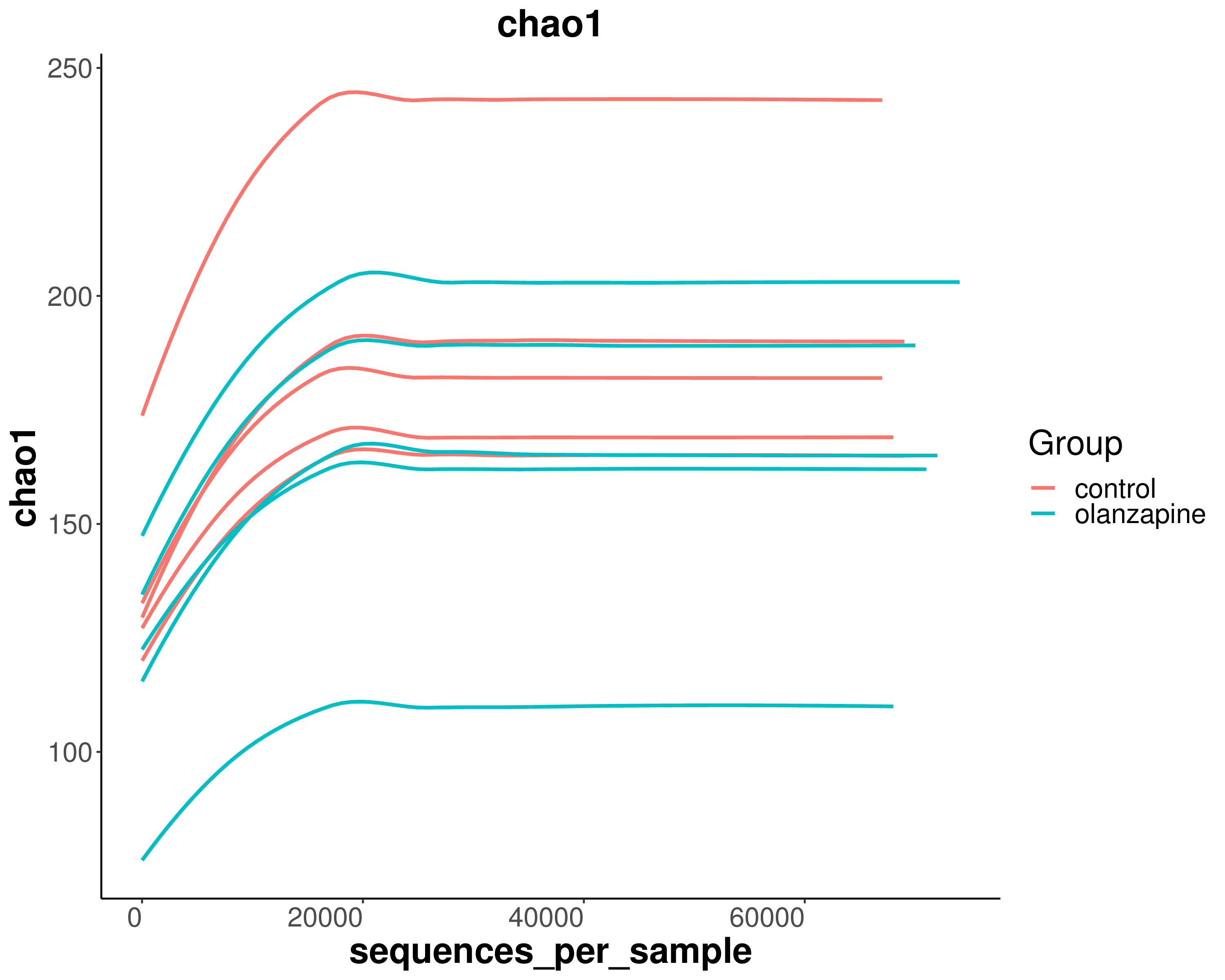

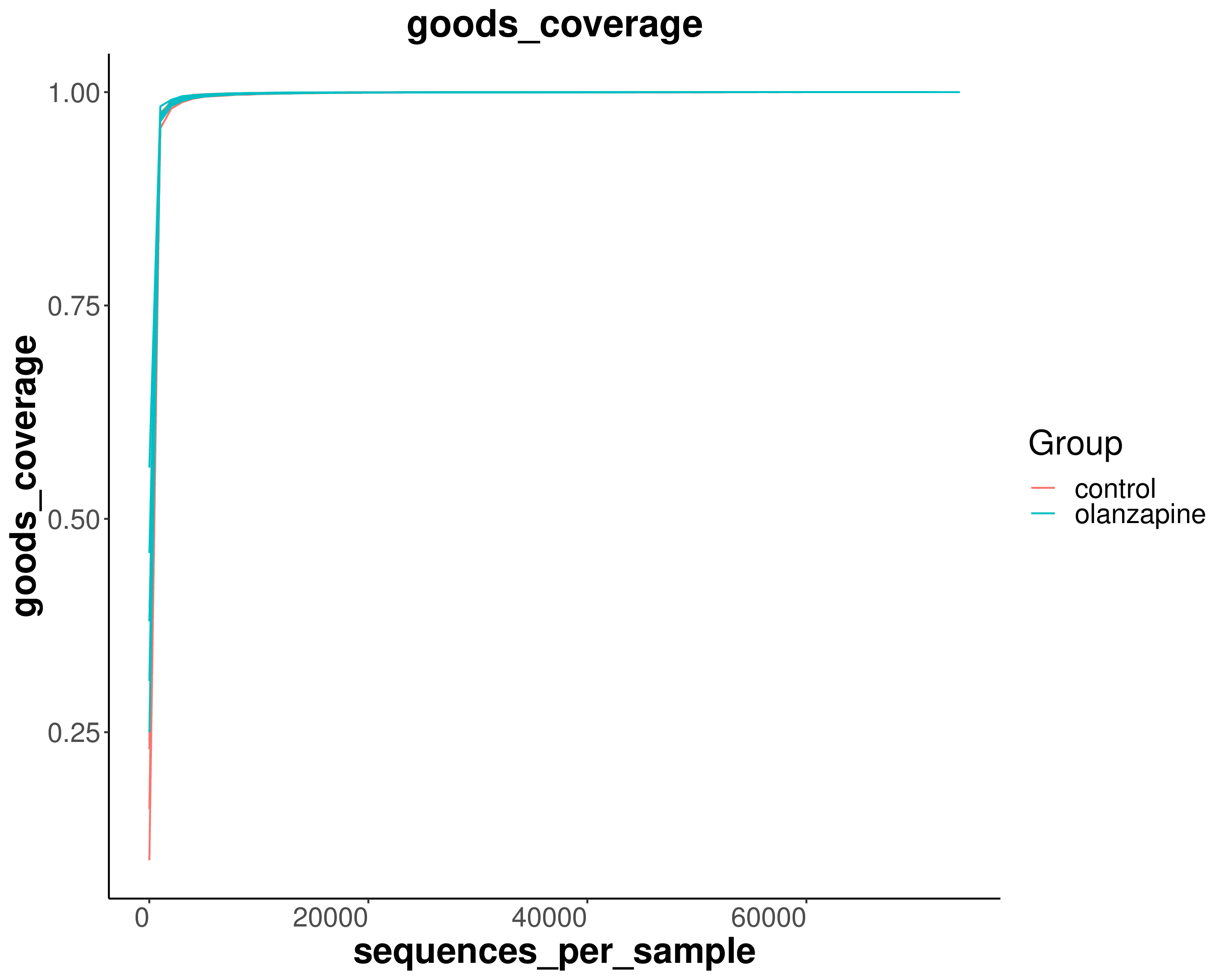

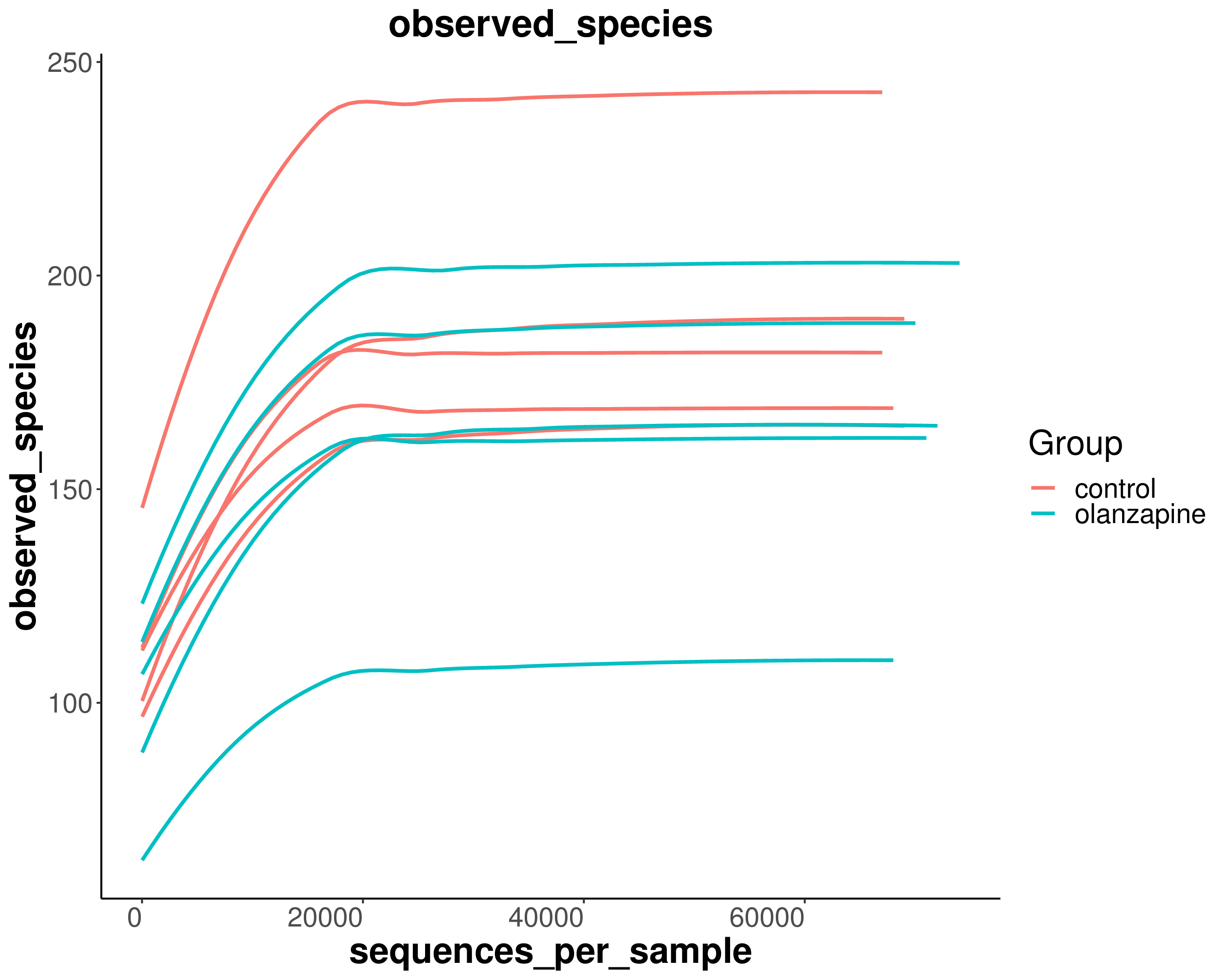

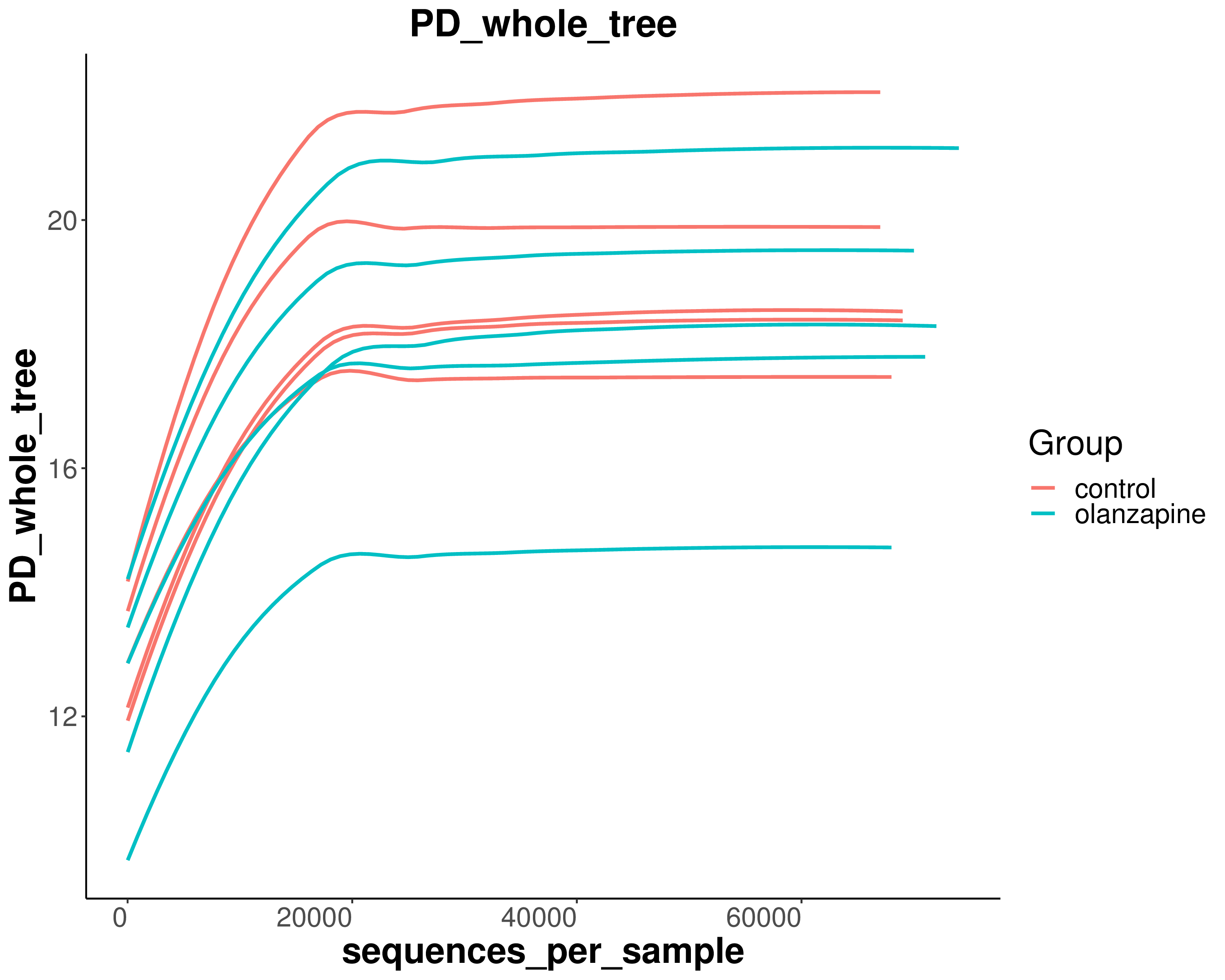


**[Eubacterium]_**
**xylanophilum_group**

**B**


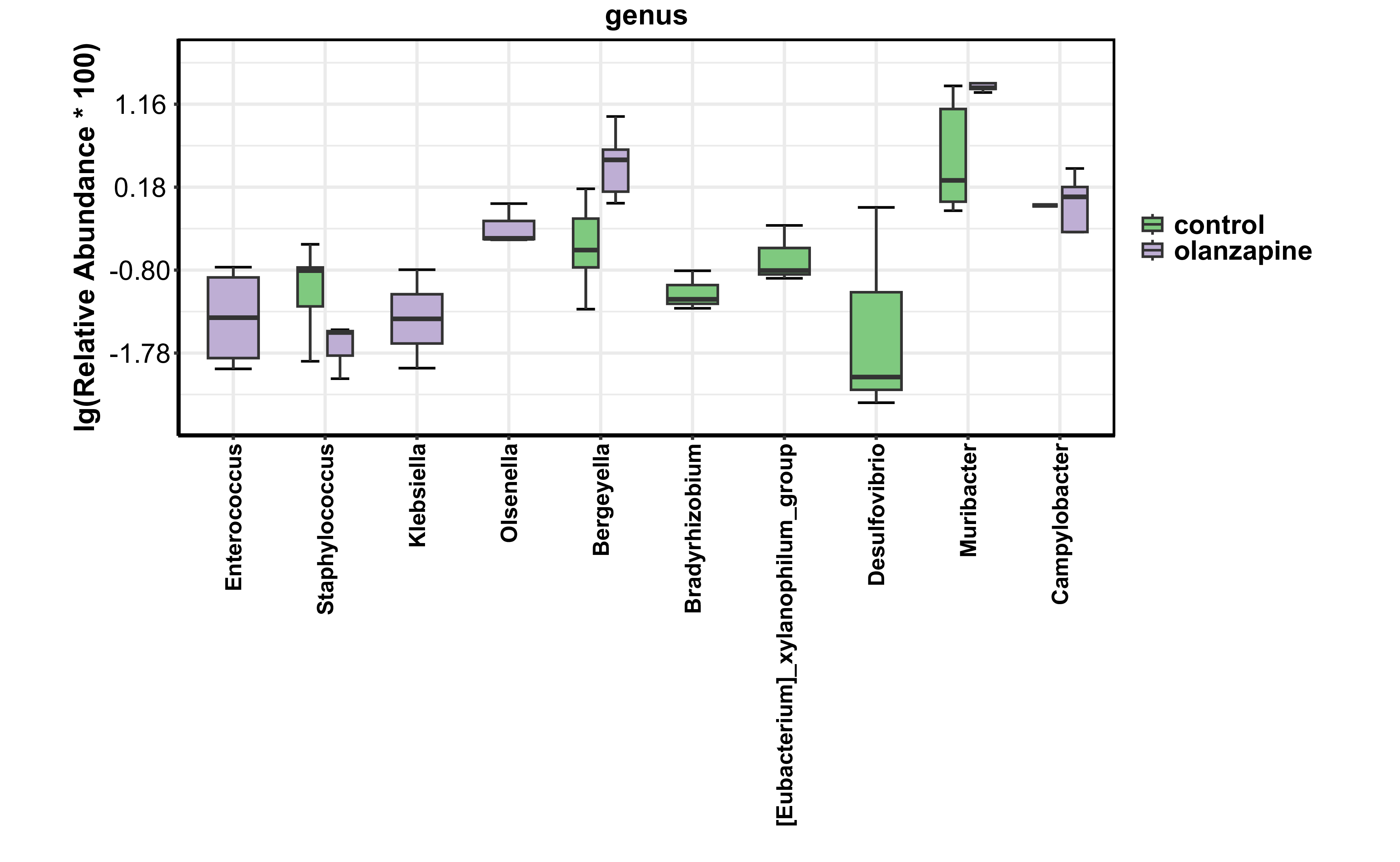

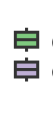


**control**

**olanzapine**

**0.18**

**1.16**

**-1.78**

**-0.80**

**Lg(Relative Abundance)*100**

**Enterococcus**

**Klebsiella**

**Staphylococcus**

**Olsenella**

**Bradyrhizobium**

**Bergeyella**

**Muribacter**

**Desulfovibrio**

**Campylobacter**

**A**


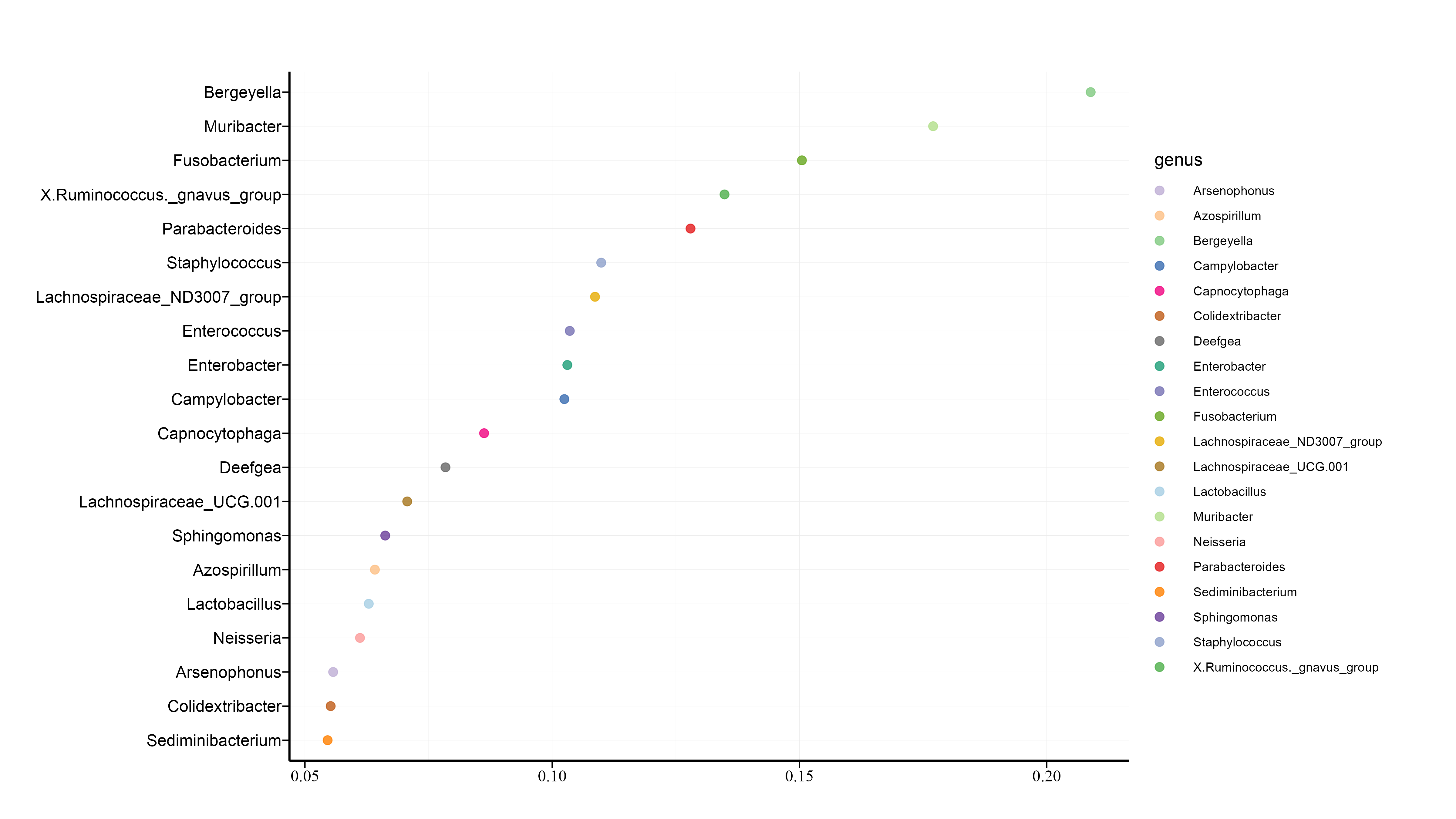


**Mean Decrease Gini**

**Genus**

**D**

**E**

**Supplementary Figure 2.** **Microbial diversity and composition analysis based on 16S rRNA sequencing.**
(**A**) Rarefaction curves showing the α-diversity indices of the control and olanzapine-treated groups, including PD whole tree, Chao1, Goods coverage, observed species, Shannon, and Simpson indices.
(**B**) Genus-level relative abundances in the control and olanzapine-treated groups. Statistical comparisons were performed using the two-sided Wilcoxon rank-sum test. For *Enterococcus*, the *P*-value was 0.005.
(**C**) Bar plot showing the relative abundance of *Enterococcus* in the control and olanzapine-treated groups.
(**D**) Top 20 random‑forest genus‑level importance in the control and olanzapine‑treated groups. *Enterococcus* ranked among the top predictive genera.

(**E**) Relative abundance of *Enterococcus* in saliva from olanzapine-treated patients and healthy controls measured by qPCR. Data are presented as mean ± SD. Statistical analysis was performed using Student’s *t*-test; **P* < 0.05.


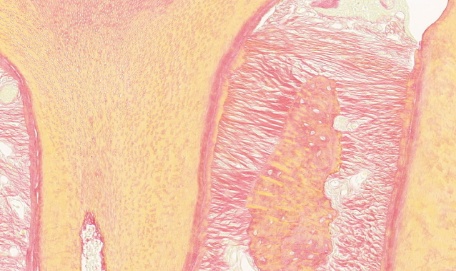

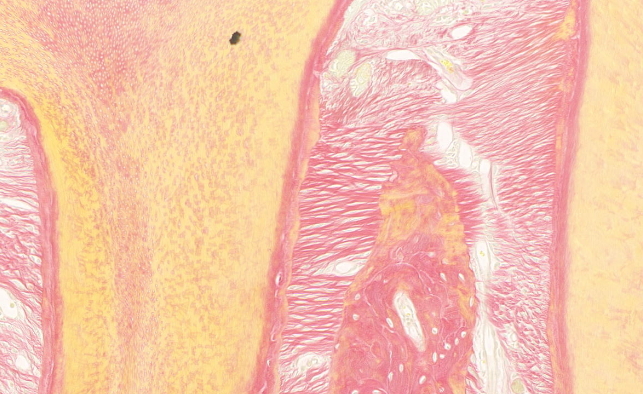

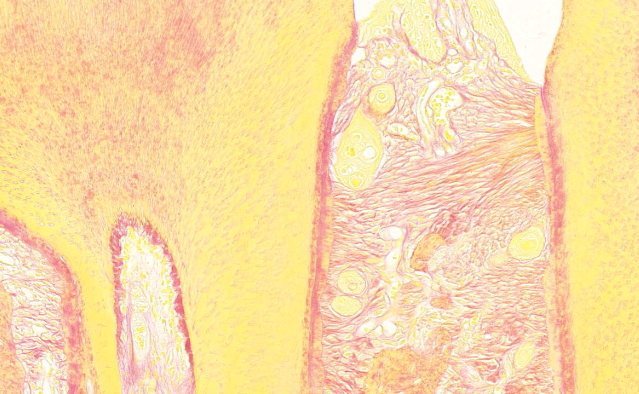


**Control**


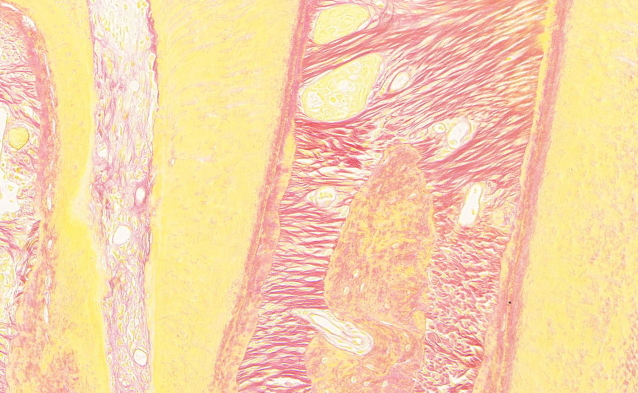


**10×**


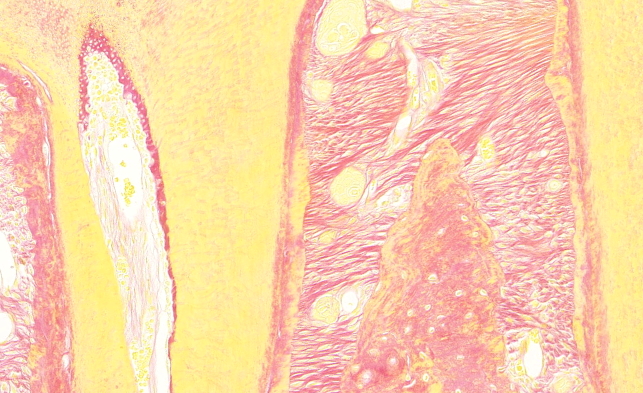

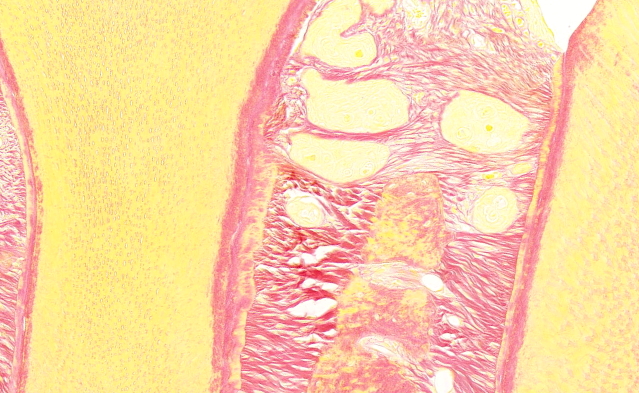


**Olanzapine**

**40×**


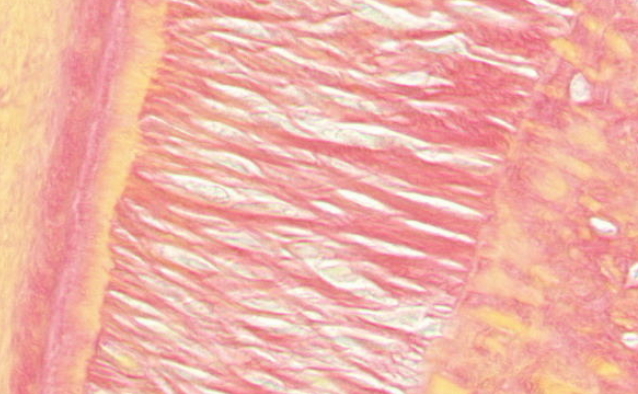

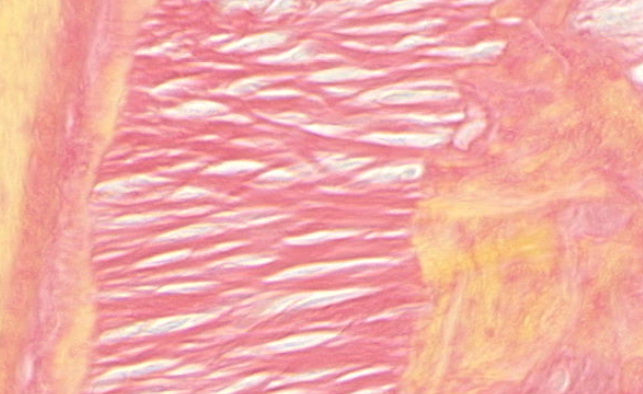

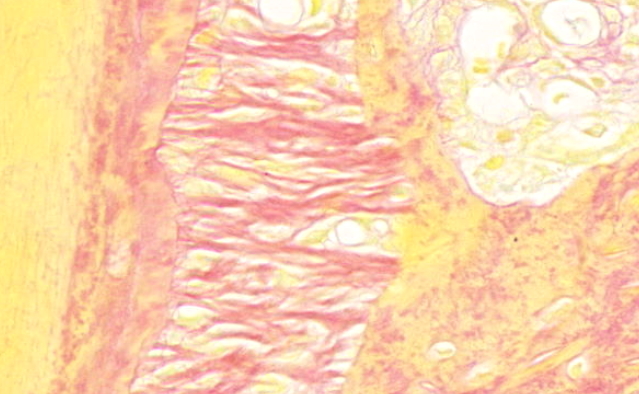

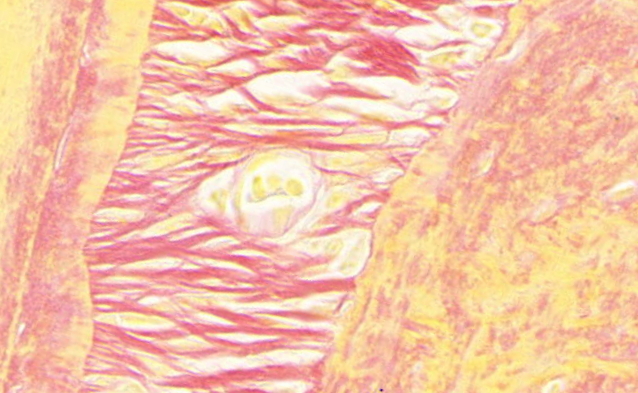

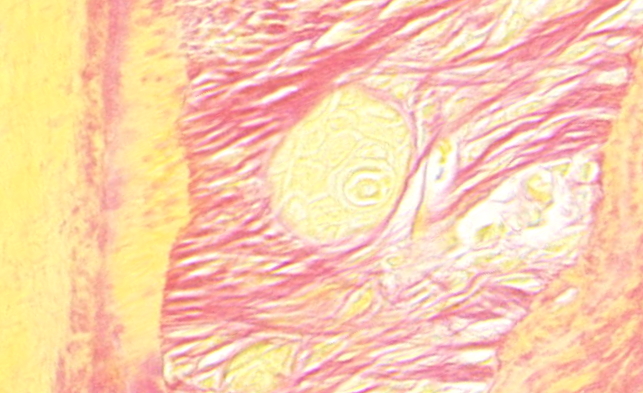

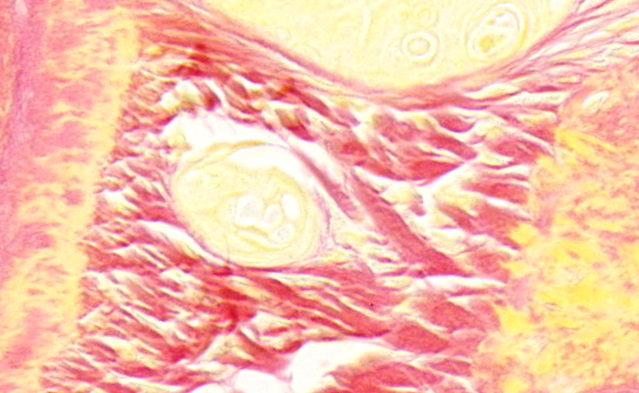


**Control**

**Olanzapine**

**Supplementary Figure 3.** **Sirius red staining of mouse periodontal tissue sections from the olanzapine-treated and control groups under bright-field microscopy.** (Representative images are shown at 10× and 40× magnifications to visualize collagen fibers.)

**Group**


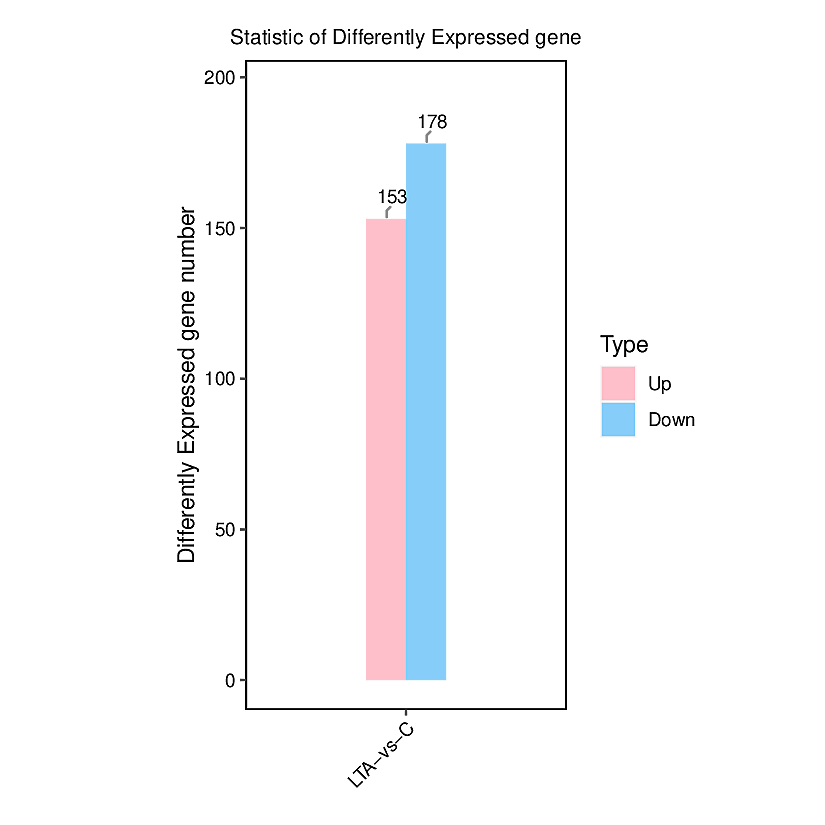


**Statistic of Differentially Expressed Genes**

**200**

**Number of Differentially Expressed Genes**

**LTA-vs-C**

**Type**

**Up**

**Down**


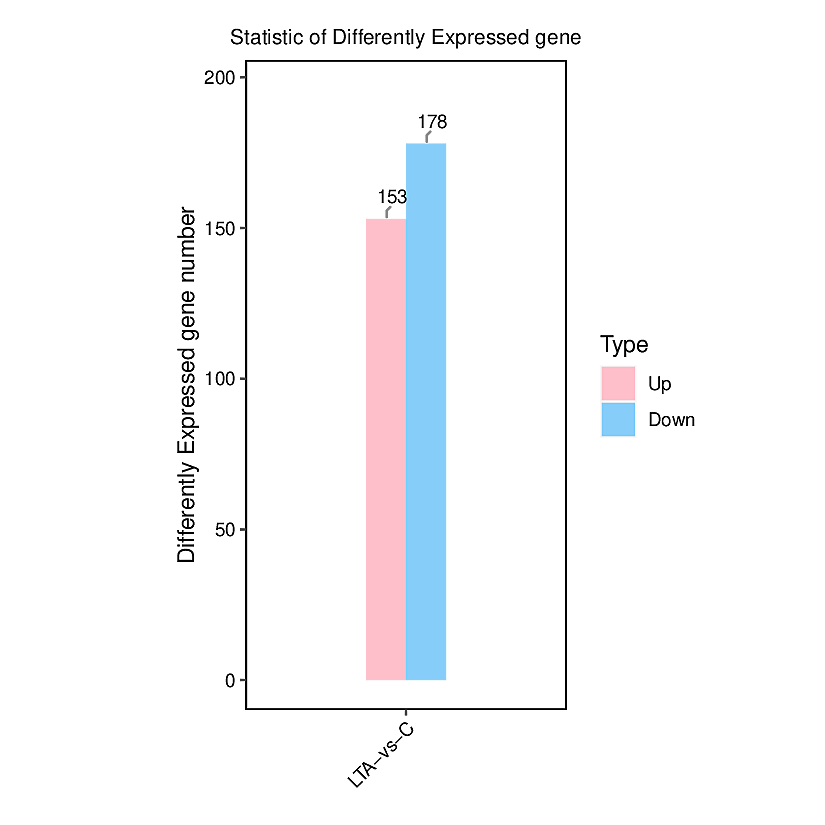


**150**

**100**

**50**

**0**

**C**


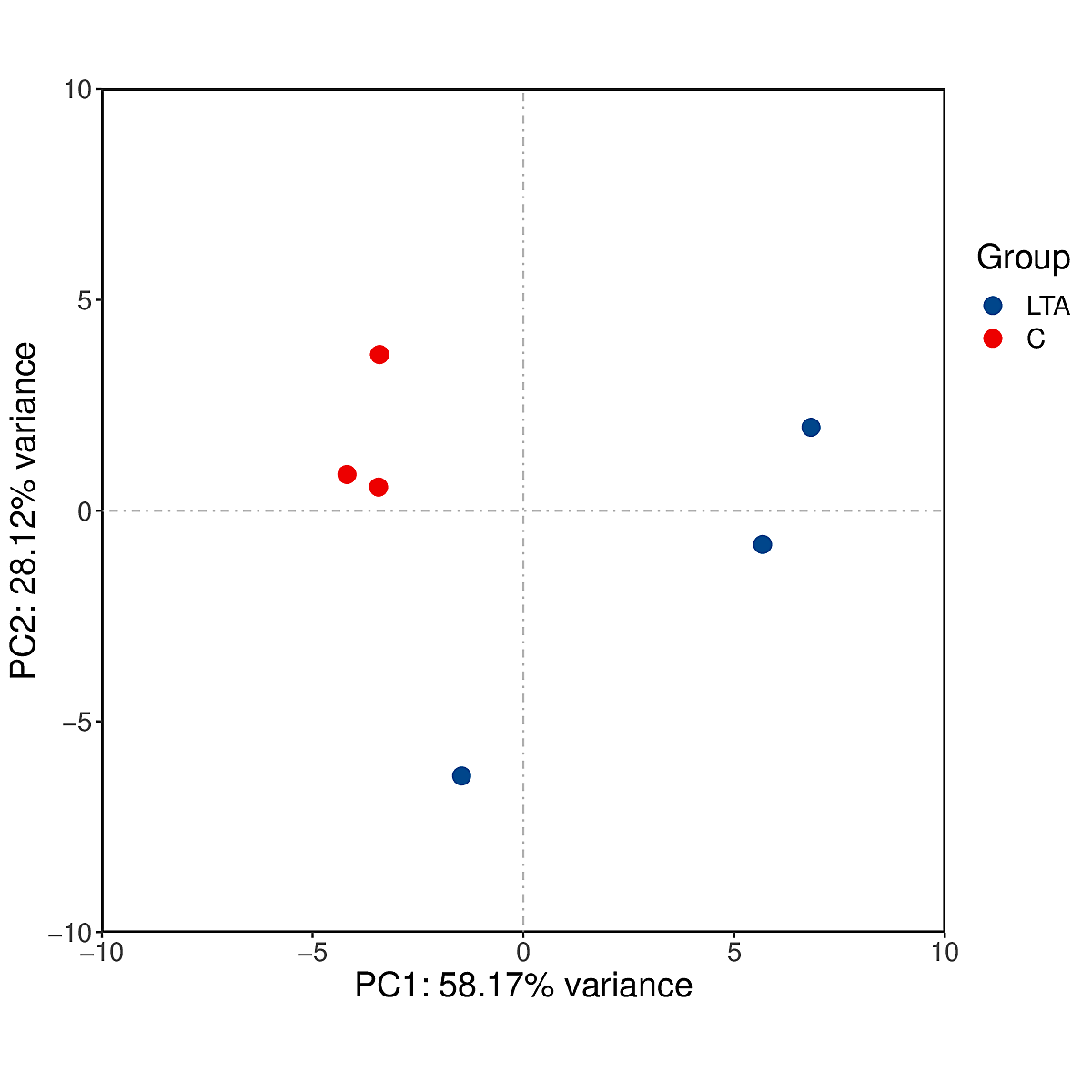

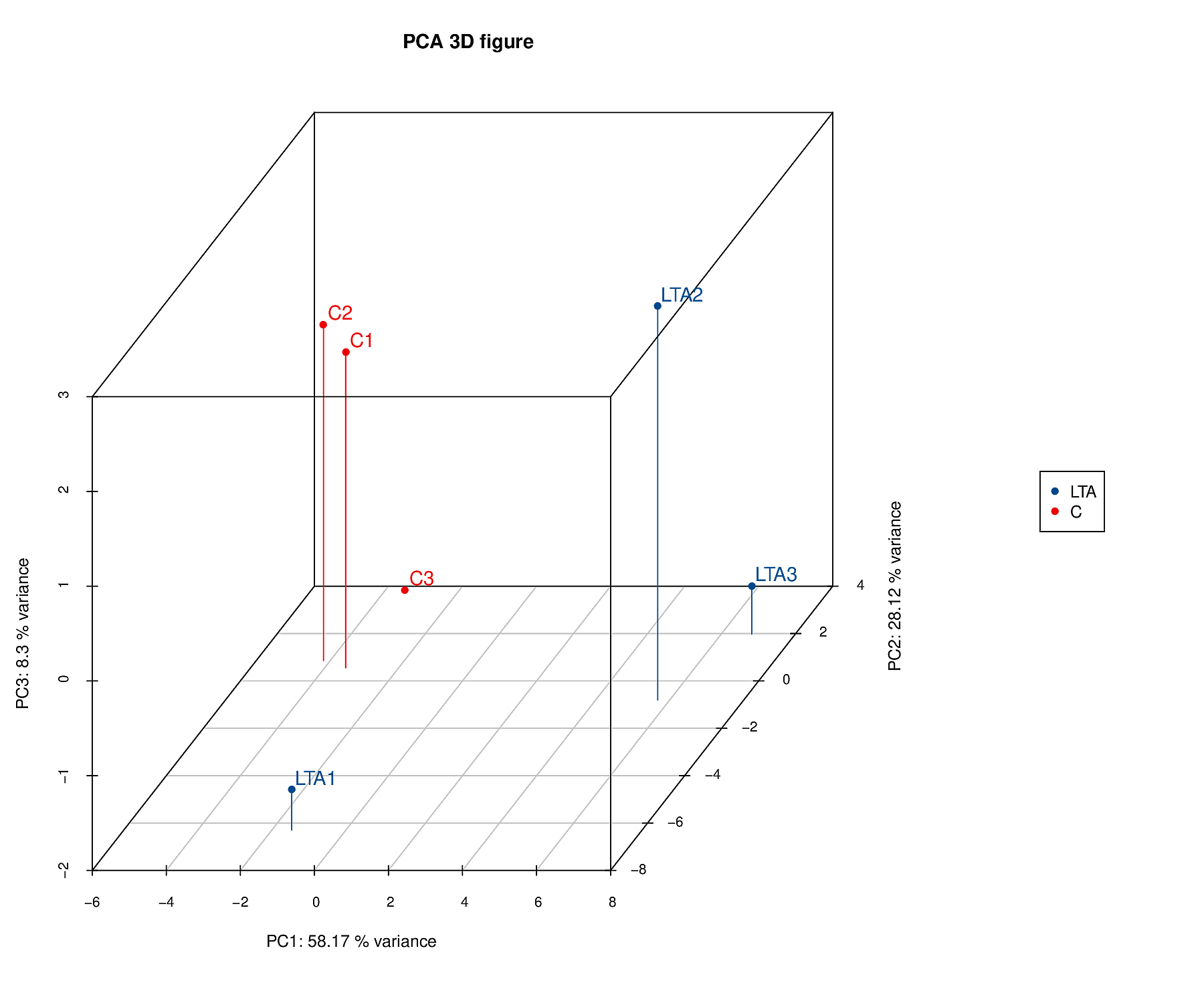


**B**

**PC1: 58.17% variance**

**PC1: 58.17% variance**

**PC2: 28.12% variance**

**PC2: 28.12% variance**

**PC3: 8.3% variance**

**LTA**

**C**

**-10 -5 0 5 10**

**10**

**5**

**0**

**-5**

**-10**


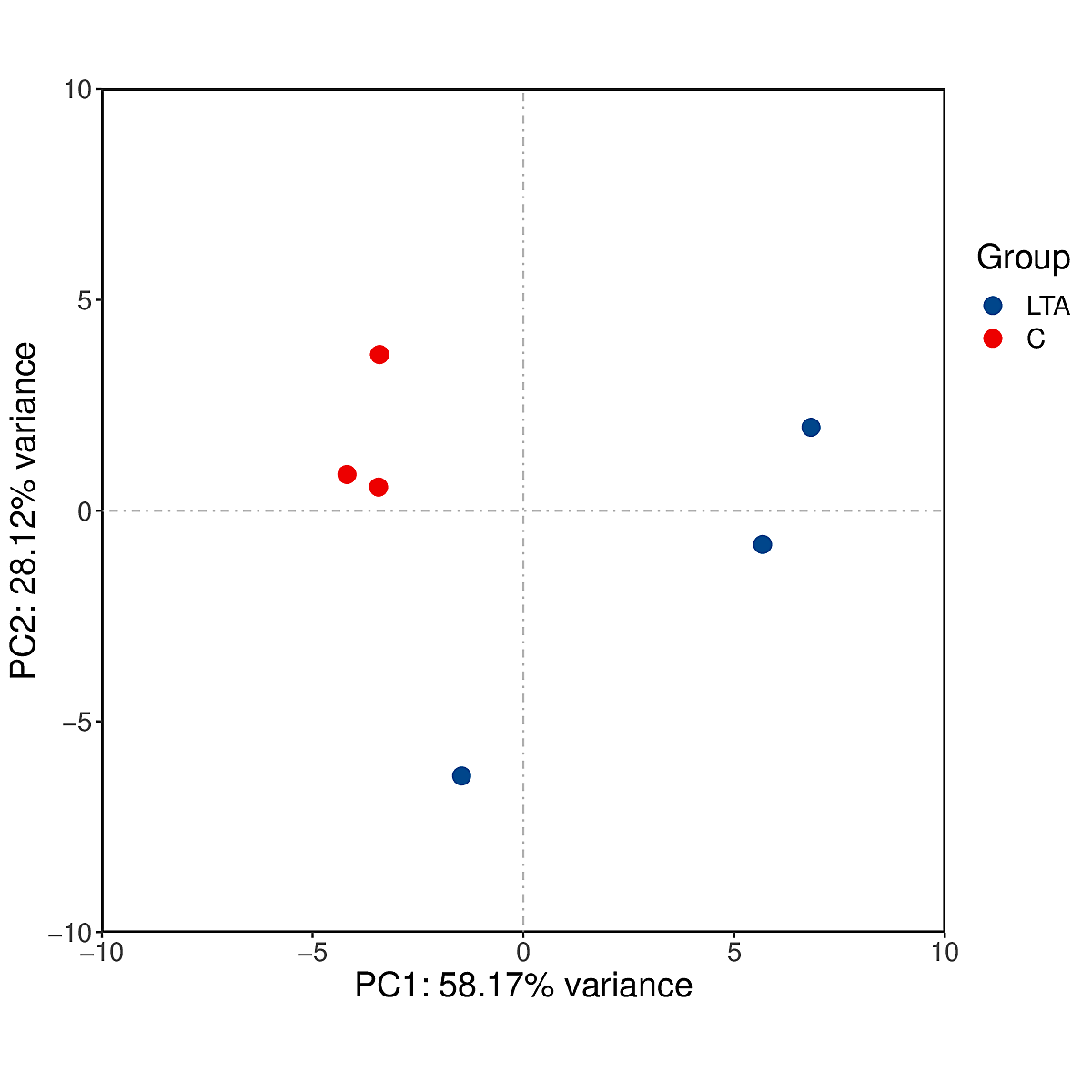


**4**

**2**

**0**

**-2**

**-4**

**-6**

**-8**

**-6 -4 -2 0 2 4 6 8**

**PCA 3D figure**

**LTA**

**C**


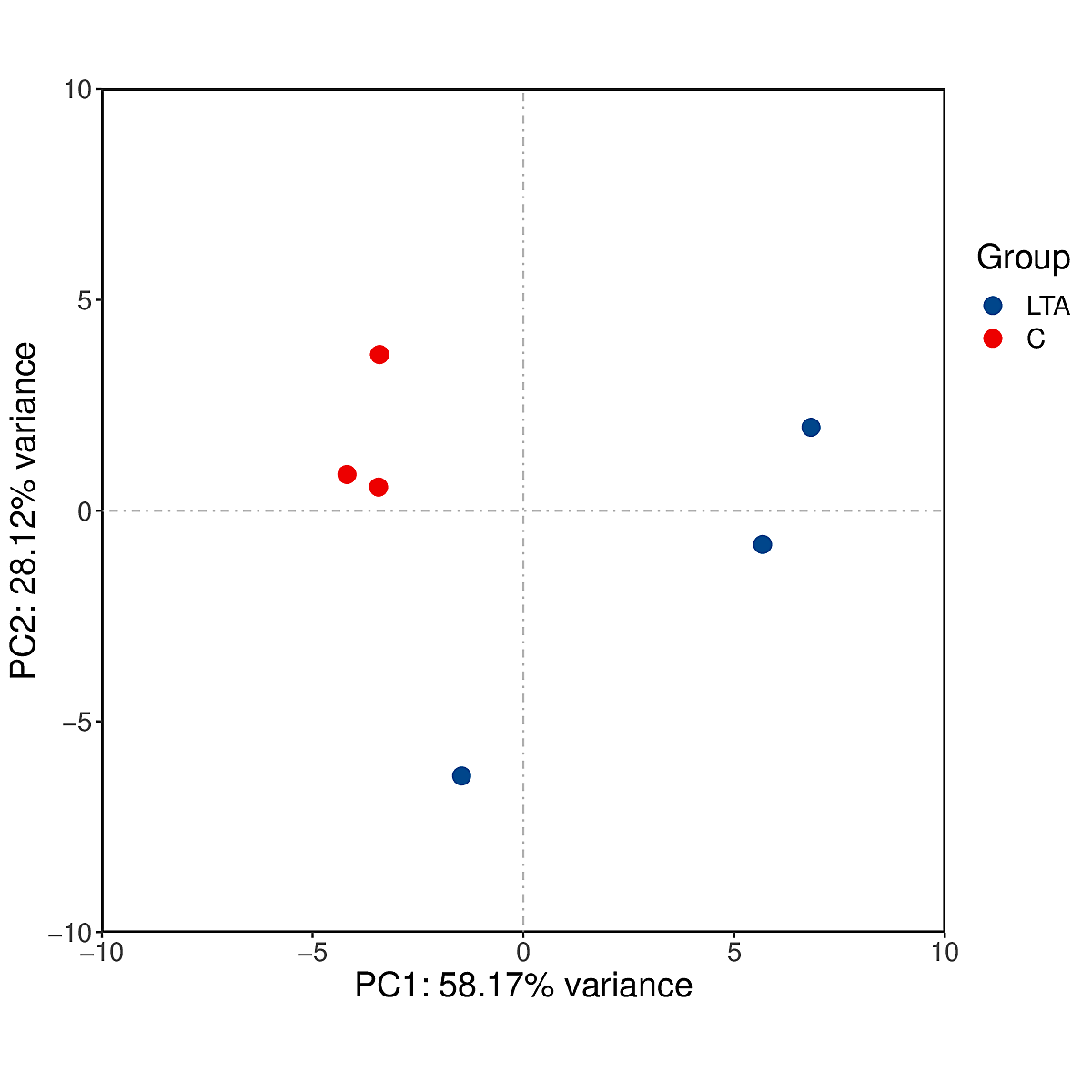

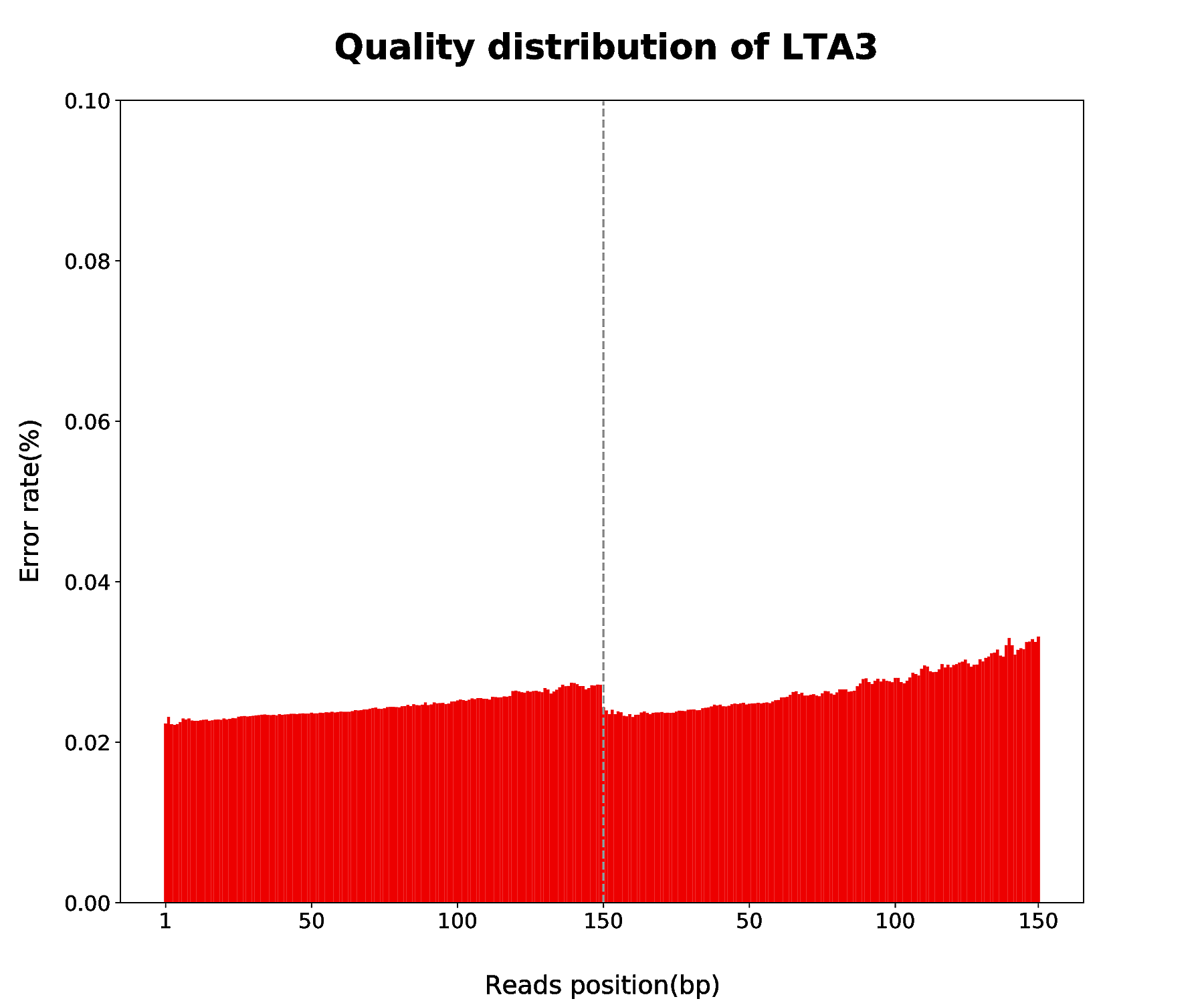

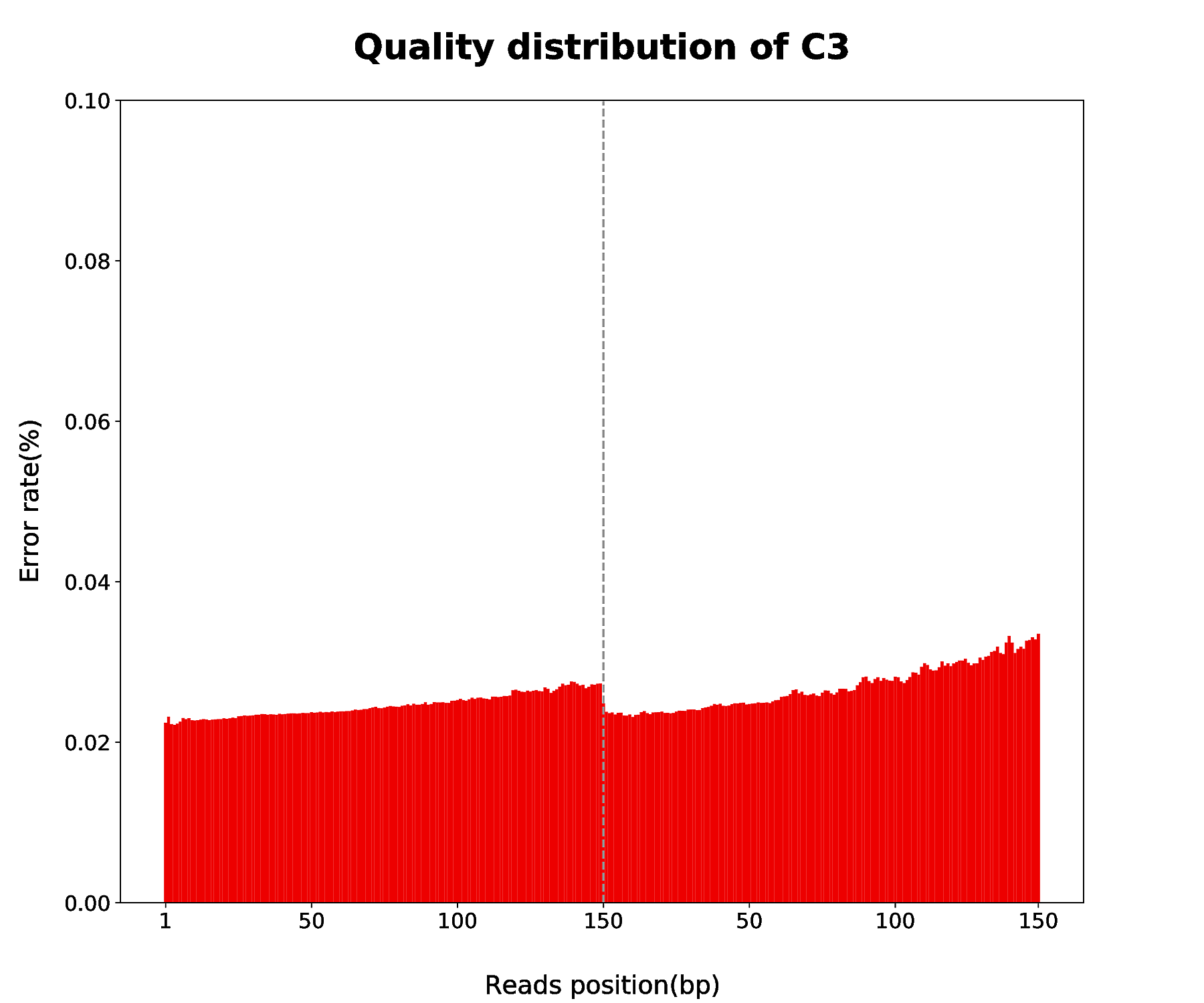

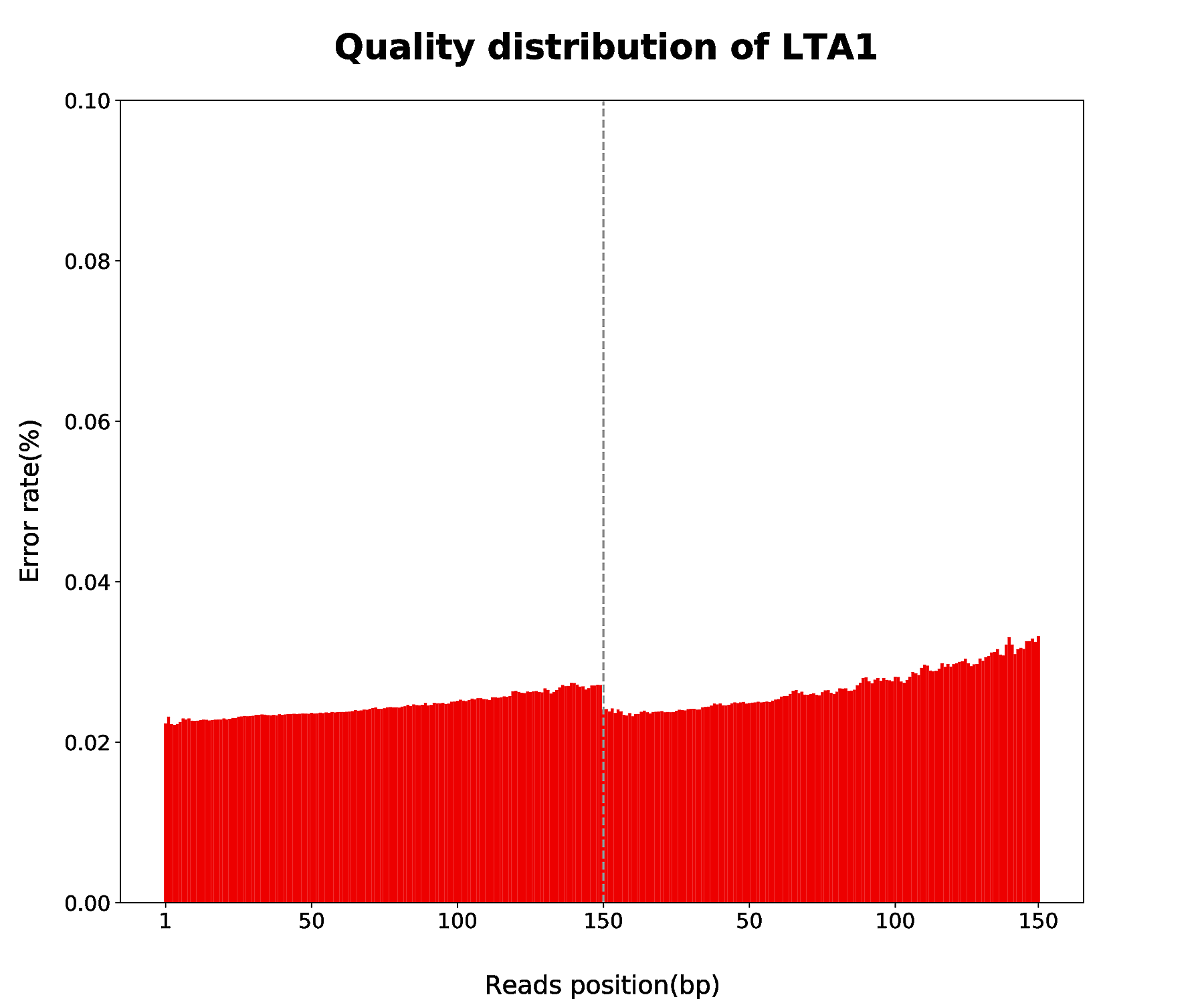

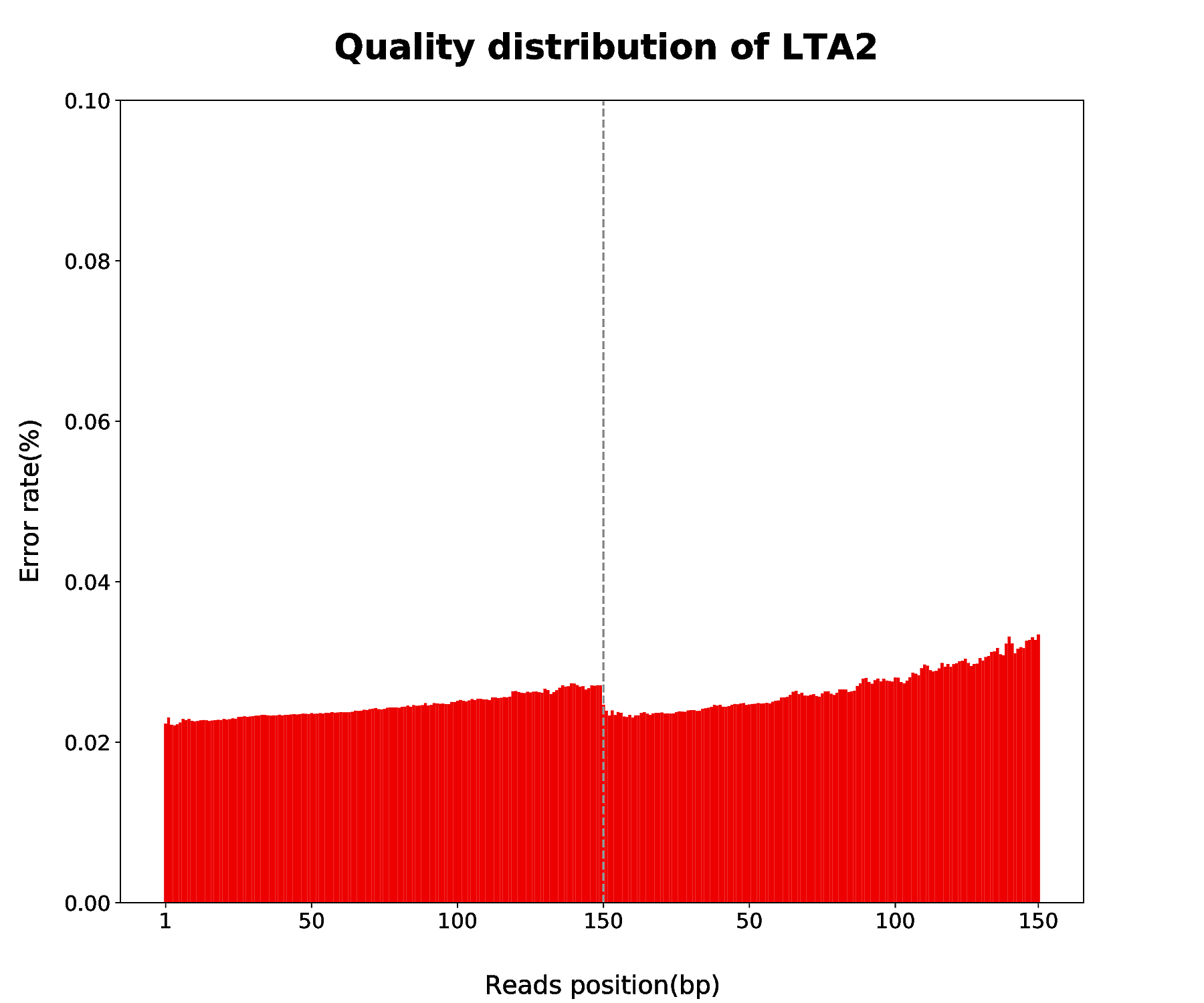

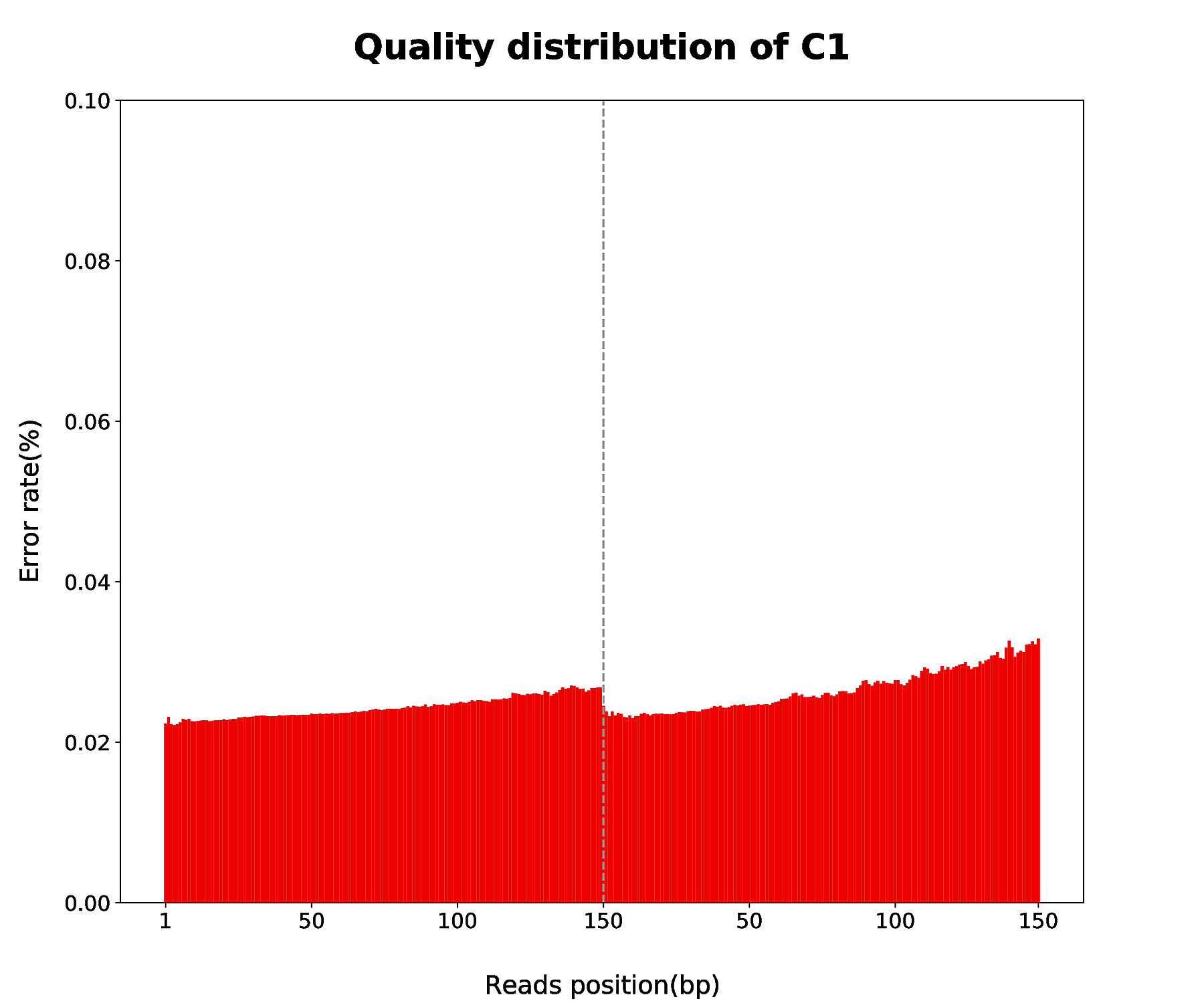

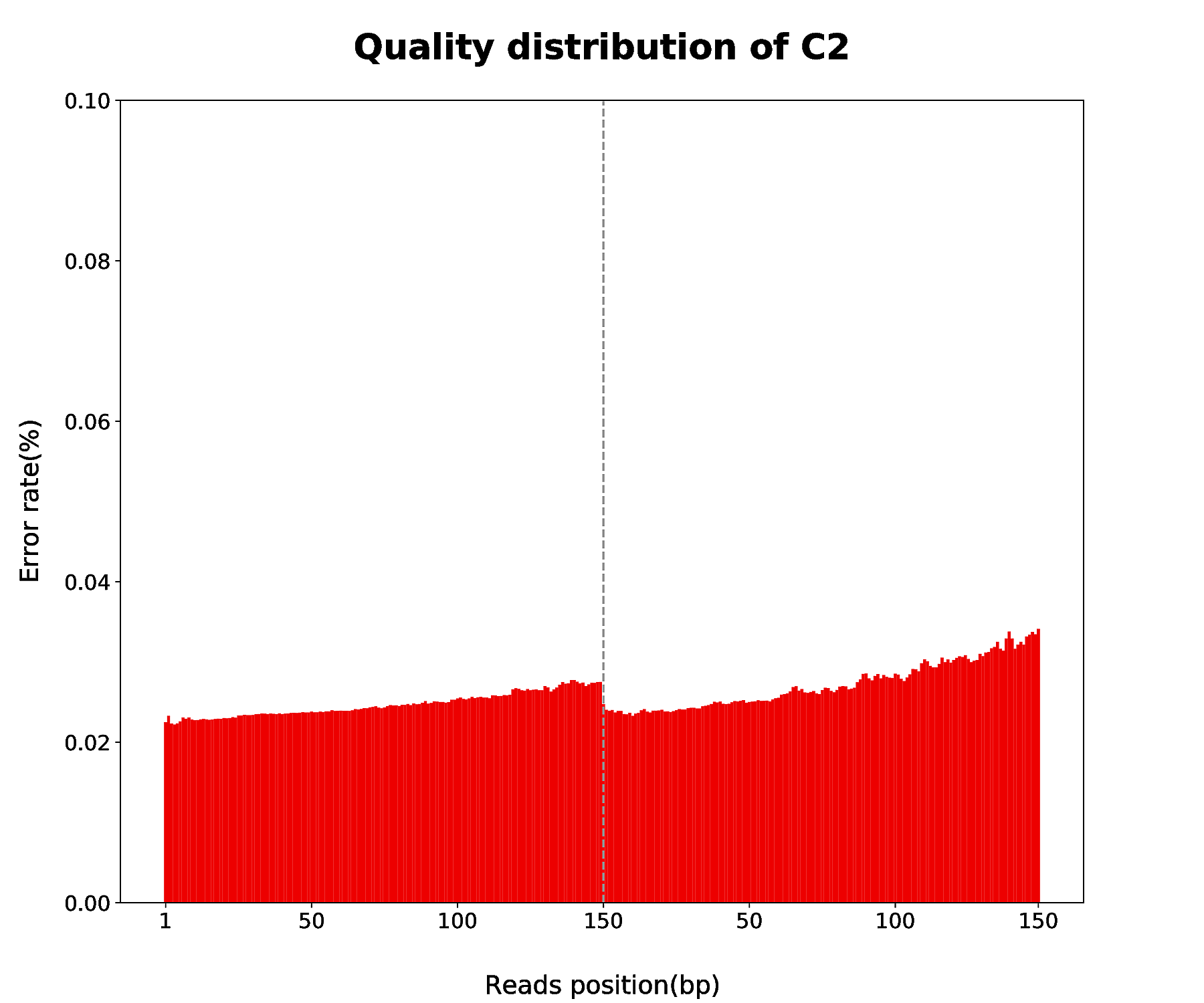


**D**

**-2 -1 0 1 2 3**

**A**

**Supplementary Figure 4.** **Transcriptomic sequencing quality control and gene expression analysis of hPDLSCs after LTA treatment.**
(**A**) Base quality score distribution plots of each sample.

(**B, C**) Two-dimensional (B) and three-dimensional (C) principal component analysis (PCA) plots showing the clustering of control (C) and LTA-treated (LTA) groups based on gene expression profiles.

(**D**) Bar plot showing the number of differentially expressed genes (DEGs) between the control and LTA-treated groups.


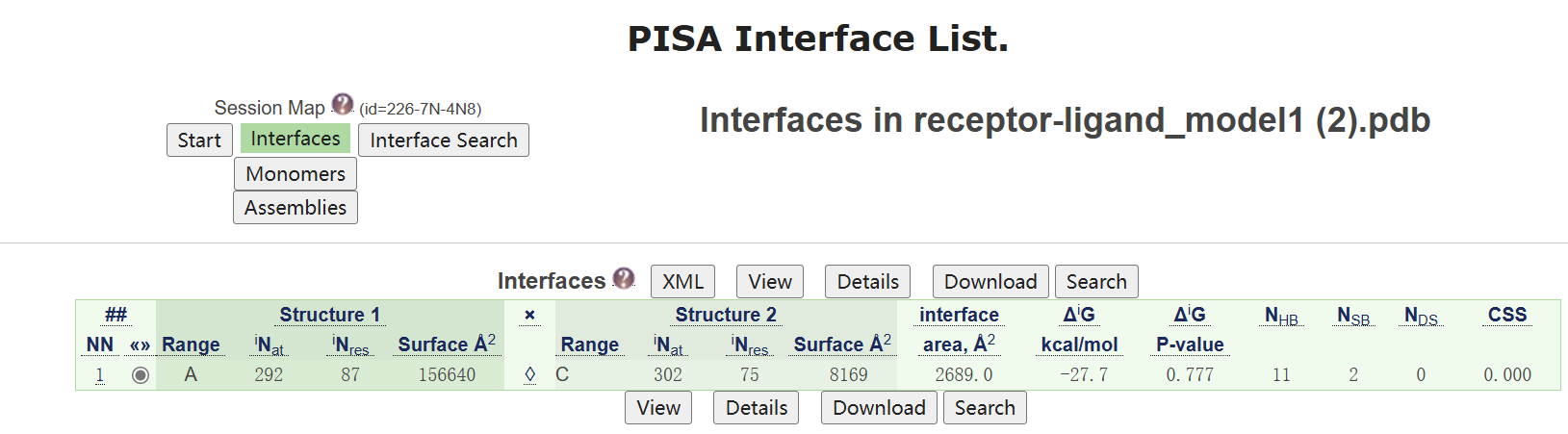

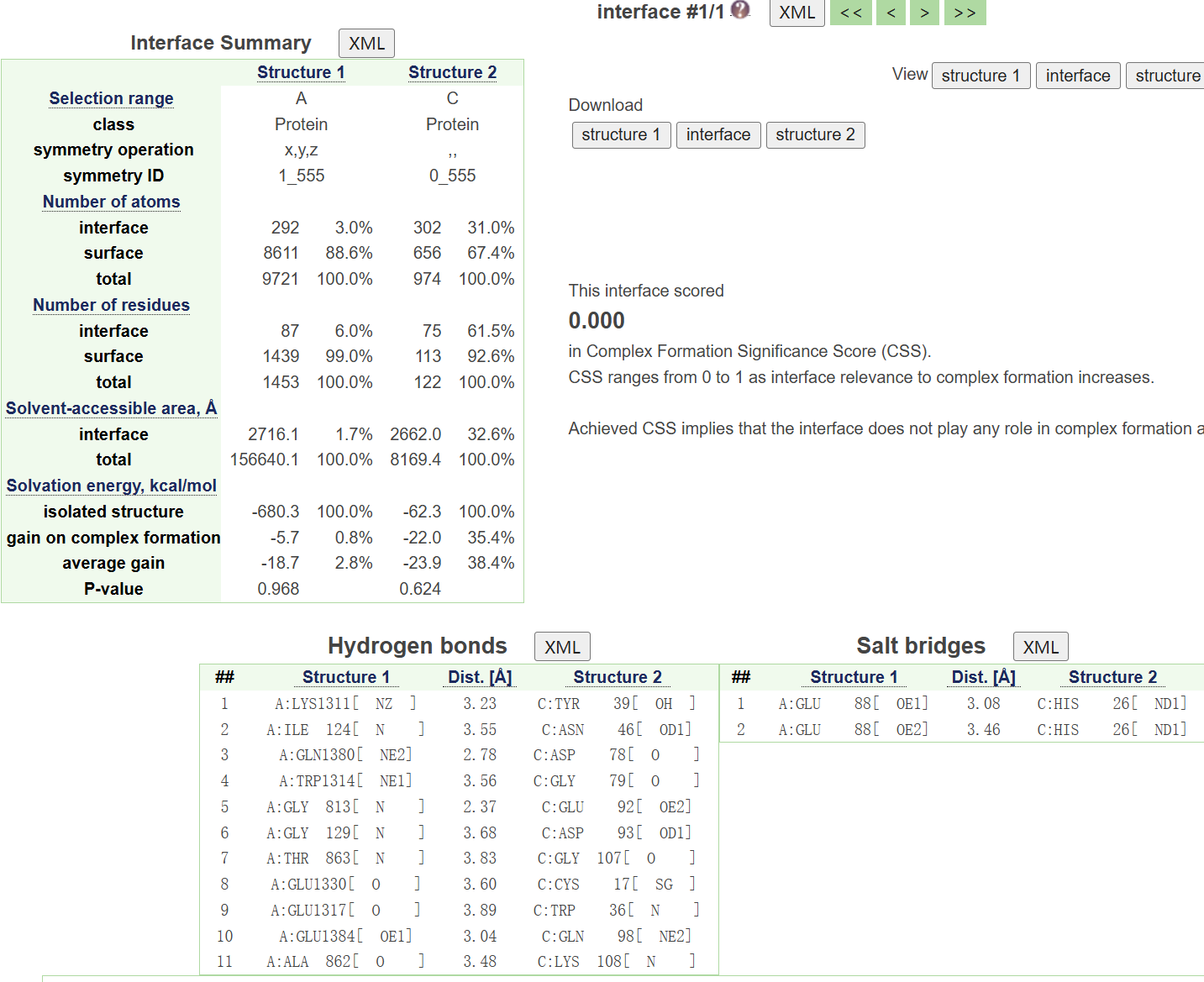

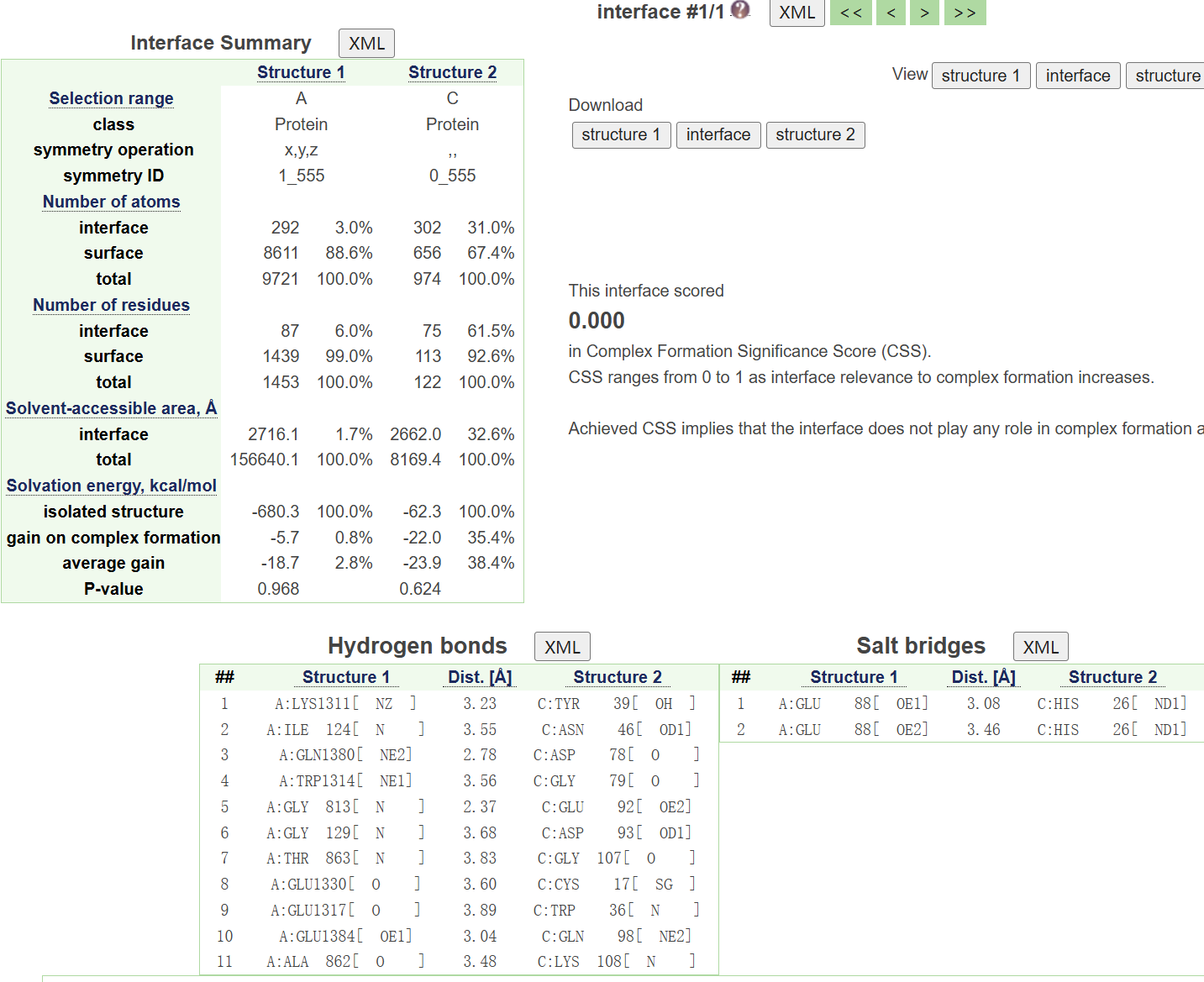


**A**

**B**

**C**

**Supplementary Figure 5.** **Prediction of interaction between SAA1 and COL1A1 proteins by PISA analysis.**
 (**A**) PISA Interface List. (**B**) Interface Summary. (**C**) Predicted hydrogen bonds and salt bridges at the interface between SAA1 and COL1A1 proteins.

# Supplementary Table

**Table S1. Association of olanzapine use with BMD Z-scores at three skeletal sites: crude and adjusted linear regression results**

| Outcome (Z-score) | Crude Model Olanzapine, β (95% CI) | *P* | Model Ⅰ Olanzapine, β (95% CI) | *P* |
| --- | --- | --- | --- | --- |
| Femoral Neck | -1.16 (-1.67, -0.65) | <0.001 | -1.34 (-1.84, -0.83) | <0.001 |
| Lumbar Spine | -0.94 (-1.43, -0.46) | <0.001 | -1.04 (-1.50, -0.57) | <0.001 |
| Hip | -0.72 (-1.17, -0.27) | 0.003 | -0.88 (-1.33, -0.43) | <0.001 |

Model Ⅰ adjusted for age, sex and BMI. β denotes the mean difference in Z-score for olanzapine users versus non-users.
